# The Immune Contexture of Liposarcoma and Its Clinical Implications

**DOI:** 10.3390/cancers14194578

**Published:** 2022-09-21

**Authors:** Antonia Resag, Giulia Toffanin, Iva Benešová, Luise Müller, Vlatko Potkrajcic, Andrej Ozaniak, Robert Lischke, Jirina Bartunkova, Antonio Rosato, Korinna Jöhrens, Franziska Eckert, Zuzana Strizova, Marc Schmitz

**Affiliations:** 1 Institute of Immunology, Faculty of Medicine Carl Gustav Carus, TU Dresden, Fetscherstraße 74, 01307 Dresden, Germany; 2 Department of Surgery Oncology and Gastroenterology, University of Padova, Via Gattamelata 64, 35128 Padova, Italy; 3 Department of Immunology, Second Faculty of Medicine, Charles University, University Hospital Motol, V Úvalu 84, 150 06 Prague, Czech Republic; 4 Department of Radiation Oncology, Eberhard-Karls-University Tuebingen, Hoppe-Seyler-Straße 3, 72076 Tuebingen, Germany; 5 Third Department of Surgery, First Faculty of Medicine, Charles University, University Hospital Motol, V Úvalu 84, 150 06 Prague, Czech Republic; 6 Veneto Institute of Oncology IOV-IRCCS, Via Gattamelata 64, 35128 Padova, Italy; 7 Institute of Pathology, University Hospital Carl Gustav Carus, Fetscherstraße 74, 01307 Dresden, Germany; 8 National Center for Tumor Diseases (NCT), University Hospital Carl Gustav Carus, TU Dresden, Fetscherstraße 74, 01307 Dresden, Germany; 9 German Cancer Consortium (DKTK), Partner Site Dresden, and German Cancer Research Center (DKFZ), Im Neuenheimer Feld 280, 69120 Heidelberg, Germany; 10 Department of Radiation Oncology, Medical University of Vienna, Waehringer Guertel 18-20, 1090 Vienna, Austria

**Keywords:** liposarcoma, immune architecture, immunotherapy

## Abstract

**Simple Summary:**

Liposarcomas (LPS) are malignancies arising from adipose tissue. Based on the histological appearance, five subtypes are distinguished: well-differentiated LPS, dedifferentiated LPS (DDLPS), myxoid LPS (MLPS), pleomorphic LPS, and myxoid pleomorphic LPS. Immune cells can infiltrate the tumor microenvironment (TME) of LPS and can either promote an efficient antitumor immune response or mediate immunosuppression paving the way for immune evasion of the tumor. The LPS subtypes display different TME characteristics and vary in regard to immune cell infiltration, ranging from the generally lowly infiltrated MLPS to the highly infiltrated DDLPS where immunological determinants predict response to novel antibody-based immunotherapy. Thus, immune cells in the TME can significantly affect response to therapy, disease progression, and patient survival. This review aims to decipher the immune contexture of LPS as well as its clinical association and highlights differences between the LPS subtypes that may have implications for the design of novel treatment strategies.

**Abstract:**

Liposarcomas (LPS) are the most frequent malignancies in the soft tissue sarcoma family and consist of five distinctive histological subtypes, termed well-differentiated LPS, dedifferentiated LPS (DDLPS), myxoid LPS (MLPS), pleomorphic LPS, and myxoid pleomorphic LPS. They display variations in genetic alterations, clinical behavior, and prognostic course. While accumulating evidence implicates a crucial role of the tumor immune contexture in shaping the response to anticancer treatments, the immunological landscape of LPS is highly variable across different subtypes. Thus, DDLPS is characterized by a higher abundance of infiltrating T cells, yet the opposite was reported for MLPS. Interestingly, a recent study indicated that the frequency of pre-existing T cells in soft tissue sarcomas has a predictive value for immune checkpoint inhibitor (CPI) therapy. Additionally, B cells and tertiary lymphoid structures were identified as potential biomarkers for the clinical outcome of LPS patients and response to CPI therapy. Furthermore, it was demonstrated that macrophages, predominantly of M2 polarization, are frequently associated with poor prognosis. An improved understanding of the complex LPS immune contexture enables the design and refinement of novel immunotherapeutic approaches. Here, we summarize recent studies focusing on the clinicopathological, genetic, and immunological determinants of LPS.

## 1. Introduction

Soft tissue sarcoma (STS) is a rare heterogeneous group of more than 80 malignancies that originate from mesenchymal tissues [1,2,3,4]. Together with gastrointestinal stromal tumors and unclassified sarcomas, liposarcomas (LPS) are the most frequent STS in adults [1,2,3,4]. According to the 2020 WHO classification, LPS are classified into five histological subtypes based on their specific immunohistochemical, microscopic, and macroscopic features, and genetic alterations [2,3,4,5,6,7]. The classification reflects wide variations in imaging appearance, patterns of disease progression, clinical behavior, and prognostic course in each LPS subtype. Understanding the variations among the five subtypes of LPS is crucial to establish the most appropriate therapeutic strategy, planning follow-up intervals, and selecting the most effective therapies for disease recurrence/progression [3]. The five subtypes of LPS comprise well-differentiated LPS (WDLPS), dedifferentiated LPS (DDLPS), myxoid LPS (MLPS), pleomorphic LPS (PLPS), and myxoid pleomorphic LPS (MPLPS) [2,3,4,5,6,8,9].

Currently, radical surgical resection, often combined with radiotherapy, is the main treatment option for localized LPS [3,4,5]. For patients with localized LPS, centralization of surgery is probably the most efficient strategy to reduce the risk of relapse and death [10]. However, some patients progress to advanced disease that is usually associated with a poor prognosis. The standard first-line treatment for unresectable or metastatic disease is systemic anthracycline-based chemotherapy [3,4]. However, the chemosensitivity of LPS remains very low [3,4,5].

Over the past decade, several new systemic therapies, such as immunotherapy, have entered phase II and III clinical trials, as both monotherapy and combination therapies for the treatment of STS [4,5,11]. Consequently, an exceptional interest concerning immunological determinants of the tumor microenvironment (TME) and their impact on disease development, patient survival, and response to therapy emerged. Hence, it was shown that the abundance of tumor-infiltrating immune cells correlates with patient survival in multiple tumor entities [12]. Especially tumor-infiltrating lymphocytes (TILs) play a key role in predicting response to immunotherapy [12,13], which was also reported for STS including LPS [14,15].

Thus, a thorough understanding of the immune contexture with its cellular, soluble, and membrane-bound components enables improvement of existing immunotherapy approaches, e.g., by efficient patient selection, as well as the design of novel treatment modalities, e.g., by identifying new drug targets. However, the TME of LPS is still poorly understood, mainly because of their low incidence and high heterogeneity. Therefore, this review gathers novel insights into the tumor immune architecture of LPS that may guide novel therapy options. Moreover, clinicopathological and genetic determinants as well as approved treatment regimens, and clinical trials based on immunotherapeutic strategies are summarized.

## 2. Liposarcoma Subtypes: Clinical, Pathological, and Genetic Characteristics

### 2.1. WDLPS

#### 2.1.1. Clinical Features

WDLPS is the most common type of LPS, accounting for over 40–45% of cases [4,9,16]. The distribution between men and women is equal, and both WDLPS and DDLPS typically occur among 50–70-year-old individuals [17,18]. WDLPS consists of slowly growing masses localized in the extremities, trunk, or retroperitoneum and, more rarely, in the paratesticular region, mediastinum, and head and neck region [16,17]. Although WDLPS is locally aggressive, it does not usually spread to distant areas [2,18]. However, WDLPS can dedifferentiate, and the risk of dedifferentiation is higher in deep-seated neoplasms in the retroperitoneum [3,9,18]. In 10% of cases, WDLPS can relapse and dedifferentiate into highly aggressive DDLPS with an average interval of 7.7 years [9,18].

WDLPS of the retroperitoneum can be very large in size and can displace adjacent tissues [3,16]. Hence, retroperitoneal WDLPS has a worse prognosis than WDLPS arising from other locations [3,9,16]. This may also be due to the fact that the retroperitoneal area is a more challenging anatomic site to be surgically approached [3,9]. WDLPS located in the extremities is commonly called an atypical lipomatous tumor (ALT) since local recurrence of the tumor has no impact on the overall survival (OS) of patients with extremity WDLPS [19]. On the contrary, WDLPS located deep within the trunk can be related to a fatal outcome and inoperable local recurrences were shown to be the most common cause of death in these patients [3,16]. Thus, patient prognosis largely depends on the tumor location [20]. Due to clinical and radiological overlap, a biopsy may be required to distinguish lipoma from WDLPS [19]. The histologic and cytogenetic analysis is recommended by clinical practice guidelines and may be essential for further therapeutic management of WDLPS [19]. 

#### 2.1.2. Pathological Determinants and Genetic Background

Histologically, WDLPS is divided into lipoma-like, sclerosing, inflammatory, and spindle cell WDLPS [16,17]. Even though these histological variants were identified, the clinical significance of these subclasses was not proven [21]. The most frequent WDLPS is the lipoma-like variant, which presents a proliferation of mature and differentially pleomorphic adipocytes [2,4,18]. Adipocytes are intersected by fibrous septa and contain single, enlarged hyperchromatic nuclei. Mono-vacuolated or multi-vacuolated lipoblasts may be present [2].

The retroperitoneum and the paratesticular area are associated with the presence of sclerosing WDLPS, which is characterized by scattered bizarre stromal cells and multivacuolated lipoblasts containing hyperchromatic nuclei [2,17,18]. These cells are present in the dense collagenous stroma. Cytologically, lipoblasts, atypical fibroblasts, multinucleated cells, adipocytes, and delicate and dispersed collagen can be evidenced [17,18].

The rarest variant is the inflammatory WDLPS, which often occurs in the retroperitoneum [16,17]. Histologically, inflammatory WDLPS has inflammatory infiltrates, including lymphoplasmacytic aggregates, atypical adipocytic cell populations, and bizarre multinucleated stromal cells [16,17]. In addition to the lymphoid and plasma cells, inflammatory WDLPS contain atypical cells with multiple or hyperlobated nuclei [17]. The nuclei of the atypical cells contain coarse chromatin and abundant ill-defined cytoplasm [4,17,18].

WDLPS and DDLPS are characterized by the presence of a supernumerary ring or giant rod chromosomes, which is the consequence of specific amplification of segment 12q13-15 [2,4,9,16,18]. The latter contains a large number of cancer-related genes that are implicated in tumorigenesis [2,4,18]. The most important genes present in this sequence are *MDM2* and *CDK4* [2,4,16,18,22]. *MDM2* is an E3 ubiquitin-protein ligase, which is a negative regulator of p53 and is amplified in almost all patients. *CDK4* is a member of the Ser/Thr protein kinase family and is a part of the catalytic subunit of the protein kinase complex for the G1/S cell cycle checkpoint. *CDK4* is usually co-amplified with *MDM2* in 90% of patients making it the second most commonly amplified gene in LPS [2,4,18]. *MDM2* protein overexpression, as well as *CDK4*, can be used to confirm the diagnosis of WDLPS and DDLPS because these markers are not expressed in benign adipocytic tumors [23,24]. In general, *MDM2* amplification is seen in 7% of human cancers and one-third of all sarcomas. For that reason, *MDM2* belongs to the most studied of all genomic aberrations in WDLPS [25]. *MDM2* and *CDK4* represent the common trait of WDLPS and DDLPS diagnosis, and immunostainings and/or fluorescence in situ hybridization (FISH) were shown to be particularly useful in this area. Principally, *MDM2* and *CDK4* examination allows differentiation of WDLPS from benign adipose tumors while in DDLPS, *MDM2* and *CDK4* evaluation help to exclude poorly differentiated sarcomas [26].

Among the other genes that are often co-amplified within the 12q13-15 amplicon are *HMG2A, TSPAN31,* and *FSR2* genes [2,4,18]. The former belongs to the non-histone chromosomal high-mobility group (HMG) protein family. HMG proteins function as architectural factors and are key components of the enhanceosome, a protein complex that contributes to the regulation of the expression of a target gene. Mutations in this gene are associated with lipomas and may play a role in adipogenesis and mesenchymal differentiation [2]. *TSPAN31* is a cell surface protein that plays a role in growth-related cellular processes, including cell development, activation, growth, and motility [2]. *TSPAN31* was shown to be clinically relevant in both WDLPS and DDLPS [27]. *FSR2* is a recently identified gene that codes for a signal transducing protein that links receptor tyrosine kinases to an amplification reported in up to 90% of WDLPS [25]. The FGFR/FSR2 pathway is currently discussed as a novel potential therapeutic target in retroperitoneal STS [28].

### 2.2. DDLPS

#### 2.2.1. Clinical Features

DDLPS is a high-grade undifferentiated sarcoma that typically transits abruptly from a WDLPS to a non-lipomatous sarcoma [3,17,18]. It accounts for 18–20% of all LPS with an age and sex distribution similar to those of WDLPS [2,4,16,18]. DDLPS usually form large-sized painless masses of at least several centimeters in diameter that commonly arise in the trunk, extremities, and retroperitoneum [4,16,17,18]. It has a well-differentiated portion that is clearly demarcated from the highly cellular, spindle cell-rich, dedifferentiated portion [2,17]. By imaging, DDLPS presents as a non-homogenous fat-containing tumor with a solid component [2,17,29]. 

However, the fatty component is not present in around 25% of the cases and the terminal differentiation to adipocytes is also impaired in DDLPS [30]. Hence, the promotion of adipogenesis in DDLPS was discussed as a potential approach to restrain tumorigenicity [31]. The lesion can be discovered incidentally and within the diagnostic process, a biopsy must be directed at both the lipomatous and non-lipomatous components of the tumor to ensure accurate diagnosis and treatment [32].

Classification of DDLPS involves a differentiation between intermediate-grade (G2) and high-grade tumors (G3). High-grade DDLPS are invasive, have a more aggressive biologic nature, and spread to distant sites with a higher frequency [33].

Disease-specific mortality was reported to be significantly higher than that of WDLPS, ranging between 30–75% [4,8,25]. This is because of high rates of local and metastatic recurrence [2,3,8,16,18]. The aggressive biological behavior of DDLPS, together with its tendency to spread to distant sites, are factors that affect patient prognosis [8,25]. DDLPS-related mortality is significantly higher in G3 tumors. The early mortality, on the other hand, reflects the lower risk of local recurrences in G3 DDLPS as compared to G2 DDLPS [33].

A larger portion of DDLPS is generated de novo, but some may also originate from precursors of WDLPS lesions [3,9,16,18]. In fact, 25–40% of patients with WDLPS ultimately manifest DDLPS as recurrence [3,8,18,25]. Compared to WDLPS, DDLPS can be morphologically heterogeneous and contain both high- and low-grade dedifferentiated components [34]. Dedifferentiation occurs more frequently in deep-sealed tumors [32]. Despite the presence of dedifferentiation, tumors can recur as pure WDLPS, DDLPS, or both [8]. However, the molecular events leading to the derivation of DDLPS from WDLPS are poorly understood [30]. In DDLPS, metastases can be observed in 20–30% of cases and typically develop in the lungs [2,8]. The presence of lung metastasis is associated with a poor outcome [3,8].

#### 2.2.2. Pathological Determinants and Genetic Background

The key histological feature of DDLPS is the presence of a polymorphous population of round or spindle cells, including multinucleated forms with occasional lipoblasts [17,18,29]. The myxoid matrix and arborizing vasculature are rarely present [17].

The genomics of DDLPS is similar to that of WDLPS and is characterized by amplification of the chromosomal region 12q13-15 [2,4,18,22,35]. In general, DDLPS is known to have a more rearranged genome than WDLPS, together with a significantly higher number of gene fusions and copy number alterations [36]. In addition to the mutations that characterize WDLPS, *YEATS4*, and *CPM* are two other genes that are sometimes co-amplified within the 12q13-15 chromosomal region of DDLPS [2,4,18]. *YEATS4* has been described as a transcriptional factor that physiologically suppresses p53 function and is involved in the oncogenesis of several types of cancers. In a large-scale genomic screening study of DDLPS cells, *YEATS4* knockdown was associated with a greater antiproliferative effect than the loss of *MDM2* expression [37]. The *CPM* gene encodes carboxypeptidase M, a protease that specifically removes COOH-terminal arginine or lysine. The *CPM* gene is involved in many biological processes, including the activation, inactivation, or modulation of peptide hormone activity, and alteration of the physical properties of proteins and enzymes [2]. Other genetic alterations that were shown to affect the genomic stability and prognosis in WDLPS are presumably changes occurring on chromosomes 3, 11, and 19 [38].

Somatic mutations are not frequent in WDLPS and DDLPS. However, DDLPS development requires the accumulation of additional chromosomal abnormalities [2,4,18]. For example, amplification of 1p32 and 6q23 is frequent in DDLPS, which has a worse prognosis [2,9,39]. Additionally, in these regions, there are some genes related to dedifferentiation, such as *ASK1*, also known as *MAP3K5* (6q23.3) and *JUN* (1p32.1) [2,4,18]. The mechanism of ASK1-mediated dedifferentiation seems to involve the inhibition of peroxisome proliferator-activated receptor gamma [2,25]. On the other hand, the genes *LIPE*, *PLIN*, and *PLIN2* are uniquely absent in DDLPS suggesting the loss of adipogenesis in these tumors [40].

Other genes linked to reduced adipocytic differentiation have been identified through LPS genomic profiling [2,4,18]. These genes undergo deletions in DDLPS and decrease genome stability, resulting in a worse prognosis for DDLPS patients [2,25]. Among these, there are tumor suppressor genes, such as *RB1* (13q14.2), *ATM* and *CHEK1* (11q22-24), and *RUNX3* and *ARID1A* (1p36) [2,25,41]. Finally, chromosomal amplicons, containing *DDR2*, *ERBB3*, *FGFR1*, and *ROS1* may play a role in tyrosine kinase-mediated oncogenicity [2,25].

### 2.3. MLPS

#### 2.3.1. Clinical Features

MLPS accounts for approximately 30% of all LPS [2,4,42,43]. Unlike the other subtypes, it usually affects younger individuals between 30–50 years of age [2,43]. However, MLPS can also occur during childhood and adolescence [2,4,42,43]. Typically, it originates in the proximal extremities with almost 75% of cases occurring in the thigh [4,42]. MLPS rarely arises from the retroperitoneum [4,42,44]. Overall, local recurrence is reported in 15–30% of cases, while disease-specific mortality ranges between 15–30% [4]. MLPS can undergo a cellular transformation, which is associated with more aggressive disease and worse clinical outcomes [3,42]. Particularly after cellular transformation, MLPS can disseminate to distant sites and form metastases in up to 40% of cases [3,4,44]. Serosal membranes (peritoneum, pleura, and pericardium), the abdominal cavity, distant soft tissues, and bones are the most common site of generalization [3]. This can occur even in the absence of lung metastases [2].

#### 2.3.2. Pathological Determinants and Genetic Background

Histologically, MLPS comprises a uniform population of small, round-to-oval-shaped, non-adipocytic mesenchymal tumor cells, and a variable number of small ring lipoblasts [2,17,42,44]. Tumor cells are located within a myxoid stroma associated with a plexiform vascular network [17,42,44]. MLPS does not usually show nuclear pleomorphism, giant cells, abundant spindle cells, or increased mitotic activity [17,42]. In a subgroup of patients, cellularity can increase with the majority of cells being round with a high nuclear-to-cytoplasmic ratio [2,42]. This is recognized as high-grade MLPS [2,42]. Round cell transformation has been associated with a significantly worse five-year OS and the presence of a round cell component in more than 5% of the cells has been shown to display a higher rate of metastases as compared to MLPS poor in round cells [21].

Patients with MLPS show unique chromosomal rearrangements: t(12;16)(q13;p11) or t(12;22)(q13;12) [2,4,18,45]. The first chromosomal rearrangement results in a fusion of *FUS* and *CHOP* (also called *DDIT3*) genes (95% of patients) [2,4,18,44]. The second, which is rarer, leads to the fusion of *EWSR1* and *CHOP* [2,4,18,44]. *FUS* and *EWSR1* belong to the FET family and are involved in RNA processing and transcriptional regulation. Contrastingly, *CHOP* is a dominant-negative inhibitor of the transcription factors C/EBP and LAP and plays a role in adipocyte differentiation. Thus, the *FUS*-*CHOP* fusion protein is thought to be tumorigenic through the dysregulation of adipocytic differentiation [2,46]. As a result, the proliferation of immature lipoblasts that are incapable of differentiating is initiated [2,46]. Moreover, gene expression studies of MLPS underlined the involvement of other genes, such as upregulation of *MET*, *RET*, and *PIK3CA*, as well as deletion of *PTEN*, a tumor suppressor gene [2,4,18]. Since nuclear-localized *CHOP* is overexpressed in all cases of MLPS, Scapa et al. suggested that CHOP immunohistochemistry (IHC) could be used as a tool for diagnosing MLPS [47]. A targeted RNA sequencing assay that utilizes the Archer Anchored Multiplex PCR technology has also been validated as a detection assay for gene fusions in solid tumors [48].

### 2.4. PLPS

#### 2.4.1. Clinical Features

PLPS is a rare type of LPS, accounting for approximately 5% of all cases [4,49]. PLPS usually develops during adulthood (>50 years old) with a slight male predominance [4,49,50]. Normally, PLPS arises in the limbs, or sometimes in the trunk or retroperitoneum [2,3,4,49]. Most PLPS are located in deep soft tissues, but 25% develop in the skin or subcutaneous tissues [4,49,50]. The typical morphology of PLPS is characterized by a variable number of pleomorphic lipoblasts on a background of high-grade pleomorphic sarcoma [2,17,49]. Local recurrence occurs in 30–45% of cases, and tumor-associated mortality is 30–35% [4,49,50,51]. Factors associated with a worse prognosis include large tumor size, high mitotic rate, truncal and deep location, and vascular invasion [2,3,4]. Furthermore, cutaneous and subcutaneous PLPS have better outcomes because they are not very aggressive and have a very low risk of metastasizing [2,4,49]. Except for the cutaneous and subcutaneous forms, PLPS tends to be the most aggressive type of LPS [2,3,4,49]. Moreover, PLPS has limited chemosensitivity and metastases can develop in up to 50% of cases. The most common sites of metastases are the lungs (75%) and liver (25%) [3,4,49,50].

#### 2.4.2. Pathological Determinants and Genetic Background

The histology of PLPS is characterized by the presence of lipoblasts in the absence of well-differentiated components [2,4,17]. The tumor cell population contains pleomorphic spindle cells, round cells, and multinucleated giant cells, which are associated with pleomorphic multivacuolated lipoblasts [2,4,17]. Spindle cells can be arranged in fascicles [2,4,17]. The cytological features of PLPS include marked pleomorphism, coarse chromatin, prominent nucleoli, and recurring mitotic figures [2,4,17].

The molecular pathology of PLPS is still poorly understood because of its complex karyotypes. PLPS contains multiple chromosomal rearrangements, including loss-of-function (LOF) and gain-of-function (GOF) [2,4]. Genome analysis has described deletion of 13q14.2-5 in half of the patients, which contains the *RB1* gene [2]. Mutations or deletions of *TP53* can be present, whereas, in other forms of LPS, *TP53* alteration is not common [2]. In some patients, *NF1*, an oncosuppressor gene, is lost, and *p14ARF*, a p53 target gene, is epigenetically silenced [2]. Tissue sections of PLPS show cells positive for smooth muscle actin, S-100 protein, keratin, and desmin [17,49].

### 2.5. MPLPS

#### 2.5.1. Clinical Features

According to the 2020 WHO classification, MPLPS has been defined as a new distinct subtype of LPS [6,7,8,52]. MPLPS was first described by Alaggio et al. in 2009 as a subtype of LPS that mainly affects young people [53,54]. MPLPS is a rare and aggressive adipocytic neoplasm that usually occurs in children and adolescents and predominantly affects females [6,7,52]. MPLPS arises in the axial region of the body, preferentially in the mediastinum, but it can also form in the thigh, head, abdomen, and back [52,53,54]. Half of the patients can have local and sometimes multiple recurrences, owing to their highly aggressive nature [6,52]. MPLPS is associated with an increased risk of distant metastases, occurring mostly in the lungs, bones, and soft tissues [52,54].

MPLPS that arises from the thorax can invade nearby structures, such as the superior vena cava, heart, trachea, pericardium, bronchi, and esophagus [6,52]. This invasion can cause various symptoms, such as wheezing, shortness of breath, cough, tachycardia, and chest pain [53]. Disease-associated death usually occurs within 40 months [6,52].

#### 2.5.2. Pathological Determinants and Genetic Background

MPLPS shares histological features with MLPS and PLPS [6,52]. However, gene fusions and amplifications, which are typical of MLPS, are missing in MPLPS [6,52]. The tumor contains an abundant myxoid matrix containing well-developed blood vessels [6,52]. The cells are round to slightly spindle cells, which are similar to the cells of MLPS [6,52]. Moreover, in these areas, scattered tumoral cells with larger nuclei and some irregularities can be observed [6,52]. The tumor also has regions containing necrosis [6,52]. Other morphological features observed in the MPLPS include prominent fibrous septation and lymphangioma-like mucin pools [6]. Thus, MPLPS shows a distinctive combination of relatively bland zones, resembling MLPS, and more cellular and atypical areas, resembling PLPS [19]. The immunophenotype of MPLPS is rather nonspecific [7].

MPLPS is not characterized by the presence of gene fusions, such as *FUS*-*CHOP*, *EWSR1*-*CHOP*, and *MDM2* gene amplification [52,53]. Zare et al. reported a case of MPLPS in a patient with Li-Fraumeni syndrome and *TP53* germline mutations [55]. The patient tested negative for *CHOP* rearrangements and *MDM2* amplifications. Gami et al. reported a case of MPLPS in an infant that exhibited strong immunoreactivity for S100 and CD34 [53]. Moreover, *MDM2* was non-reactive.

Additionally, in a study by Creytens et al., IHC revealed diffuse CD34 and p16 expression [6]. Subsequent FISH analyses allowed the identification of *RB1* monoallelic deletions and the absence of *MDM2* amplification and *CHOP* rearrangements. Moreover, genome-wide copy number profiling of eight patients revealed a complex genetic profile with several LOF and GOF variants [6,52]. Particularly, there was recurrent GOF in chromosomes 1, 6–8, and 18–21, and recurrent LOF in chromosomes 13, 16, and 17. Losses were frequent in 13q14, which contains *RB1*, *RCTB2*, *DLEU1*, and *ITM2B* genes.

**Figure 1:**
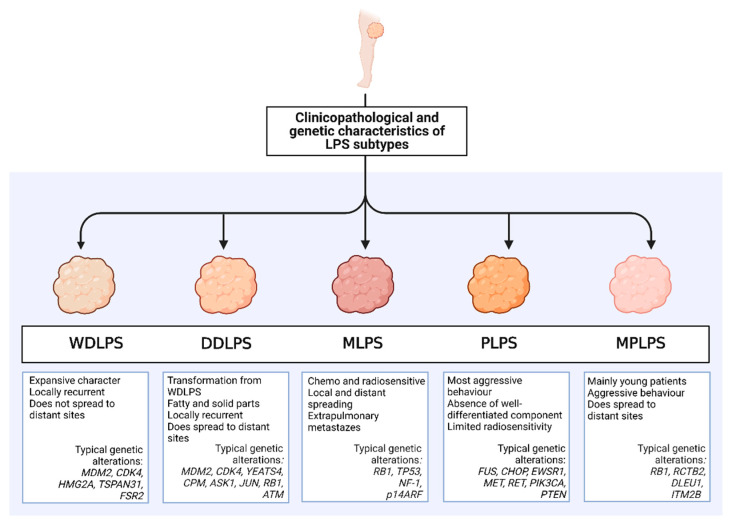
Characteristics of diverse liposarcoma (LPS) subtypes. Five distinct histological subtypes of LPS, including well-differentiated LPS (WDLPS), dedifferentiated LPS (DDLPS), myxoid LPS (MLPS), pleomorphic LPS (PLPS), and myxoid pleomorphic LPS (MPLPS) differ in clinicopathologic features, such as biologic behavior and patterns of disease progression. Genetic alterations in each subtype also display wide variations (Created with Biorender, Agreement No. JR24ANFF0H).

A summary of important clinicopathological and genetic features of the different LPS subtypes is shown in Figure 1.

## 3. Current Clinical Management and Treatment of LPS

Clinical features and standard treatment differ for WDLPS, DDLPS, PLPS, and MLPS, whereas no specific recommendations have been developed for MPLPS. In addition to differing treatment concepts, the response to standard therapy also varies between LPS subtypes. Radiologically, WDLPS hardly ever shows a reduction in size after radiotherapy which is rarely used in this entity. DDLPS and PLPS show inconsistent responses to radiotherapy and chemotherapy in standard imaging. Moreover, changes in tumor size after neoadjuvant treatment do not necessarily correlate with pathological response. In contrast, MLPS has been known for its exceptional radio- and chemosensitivity, reflected in size reduction as well as good pathological response rates after radiotherapy and chemotherapy.

### 3.1. WDLPS

Treatment for WDLPS is mostly limited to wide surgical resection with negative surgical margins. Most superficial tumors are cured by this approach and do not need multimodal treatment [56,57,58]. As these tumors usually do not metastasize [18], staging consists of local imaging, mostly magnetic resonance imaging (MRI). 

In selected cases, such as retroperitoneal LPS, neoadjuvant radiotherapy might be considered as it has been shown to thicken the tumor pseudocapsule and thus, may increase the chance of complete resection [33]. On the other hand, adjuvant radiotherapy in retroperitoneal LPS has been abandoned due to the absence of any apparent clinical benefit. Moreover, delivering postoperative adjuvant radiotherapy was associated with significant morbidity [33].

### 3.2. DDLPS and PLPS

Standard local treatment for DDLPS and PLPS of trunk and extremity includes wide resection with additional radiotherapy or amputation if limb salvage is not possible [59]. Staging encompasses local imaging with MRI and chest computer tomography (CT) to exclude pulmonary metastases. Neoadjuvant or adjuvant radiation therapy shall be offered to patients with high-grade LPS with additional risk factors (tumor size >5 cm, deep localization to superficial fascia, or inadequate surgical margins) [59,60]. Neoadjuvant treatment is increasingly used, as it has been described to decrease long-term side effects such as joint stiffness and fibrosis, in spite of higher rates of wound healing complications [61]. Additional radiation therapy may be avoided in small high-grade tumors (<5 cm) resected with good surgical margins [60]. Additional chemotherapy in the curative setting seems to prolong progression-free survival (PFS) but not OS [62]. The standard regimen in patients selected for additional chemotherapy is doxorubicin/ifosfamide. A randomized trial comparing this standard regimen to histotype-tailored chemotherapy failed to show a benefit for the stratified treatment [63]. Summarized, additional adjunctive chemotherapy in localized LPS should be considered in young patients with large (>5 cm) and high-grade LPS [60,64], as well as patients with borderline resectable tumors [65]. However, the decision about adjunctive therapy modalities should be made in multidisciplinary meetings considering the individual subtype-related chemosensitivity [65].

The role of radiotherapy is less defined in retroperitoneal DDLPS. A randomized trial did not show a benefit for radiotherapy plus surgery compared to surgery alone [66]. However, Callegaro et al. demonstrated a benefit in a propensity score matched comparison of patients treated at recruiting centers on and off trial [67]. Usually, radiotherapy is administered preoperatively, as adjuvant radiotherapy doses are hardly applicable for this anatomical location without violating normal tissue constraints.

### 3.3. MLPS

The clinical behavior and therapy response of MLPS differ from other LPS and other STS. As the metastatic pattern includes soft tissue and bone metastases, staging should be performed using whole-body CT or MRI [68,69]. Basic treatment principles are similar to DDLPS. Wide resection is often complemented with radiotherapy and chemotherapy. A distinctive trait of MLPS is the high chemo- and radiosensitivity [64,70], leading to significant size reduction after neoadjuvant treatment [71]. Thus, in addition to increasing local control, neoadjuvant treatment can also lead to better resectability in complex anatomical locations [71].

### 3.4. Additional Treatment Modalities

STS has become the tumor entity with the highest level of evidence for the addition of locoregional hyperthermia to multimodal treatment. Keeping the tumor at temperatures of 40–42 °C over one hour twice during every chemotherapy cycle with electromagnetic fields improved disease-free survival (DFS) and disease-specific survival (DSS) [72]. The biological mechanisms include improved tumor oxygenation and perfusion, inhibition of DNA repair, and immune mechanisms such as the release of danger signals [73,74].

In selected cases of locoregional disease widespread in one limb or pre-irradiated recurrences, hyperthermic isolated limb perfusion can be performed. After blocking the blood flow from and to the core of the body, the extremity is perfused with cytotoxic and/or immunologically effective substances (e.g., melphalan and tumor necrosis factor-alpha). The treatment can be applied as monotherapy or in a neoadjuvant approach [75].

### 3.5. Palliative Treatment for Inoperable and Metastatic Disease

Palliative systemic therapy for inoperable patients consists of chemotherapies such as doxorubicin monotherapy or doxorubicin with ifosfamide [76]. Other therapeutic options include trabectedin [77] as well as targeted agents such as pazopanib [78]. MLPS respond especially well to trabectedin [79]. There are a number of clinical trials with different compounds (chemotherapy, targeted therapy, immune checkpoint blockade, cellular therapies) [80]. Immune checkpoint inhibitor (CPI) therapy is evaluated as a single or combined treatment in the palliative as well as the curative setting [81].

## 4. LPS-Infiltrating Immune Cell Subsets and Their Clinical Significance

The tumor immune microenvironment is composed of cellular and non-cellular components, which are both involved in the complex tumor-immune cell bidirectional crosstalk. The former includes tumor-infiltrating immune cells that, depending on their abundance, localization, phenotype, and functional orientation, can shape the TME by either promoting efficient tumor elimination or enabling immune evasion. This section gathers novel insights into the immune infiltration of LPS as well as its prognostic and predictive value with a particular emphasis on the differences between LPS subtypes. 

### 4.1. T Cells

As drivers of cell-mediated adaptive immunity, T cells comprise several subsets mediating a variety of functions with potentially opposing effects. Subsets of particular prognostic and/or predictive value are CD8^+^ cytotoxic T cells and FoxP3^+^ regulatory T cells (Tregs), a subtype of CD4^+^ T helper (Th) cells. CD8^+^ T cells exhibit potent cytotoxic properties and are therefore crucial for facilitating antitumor immunity. Accordingly, a link between high CD8^+^ T cell infiltration and a favorable patient outcome was reported in several tumor entities [12,82]. In contrast, Tregs physiologically exhibit immune suppressor functions to maintain homeostasis and prevent an excessive immune response. Therefore, a protumoral role was proposed but the prognostic value of intratumoral Tregs remains controversial due to contradictory findings [12,82]. Besides Tregs, the infiltration of other CD4^+^ T cell subsets has been less intensively studied as clear discrimination of intratumoral Th cells remains methodologically challenging. Of these subsets, Th1 cells are largely associated with antitumoral properties while Th2 cells seem to mediate protumor effects in the TME [12]. 

Several studies indicate that T cell infiltration levels are highly variable among LPS subtypes. In general, STS exhibit a wide range of cytogenetic alterations allowing broad discrimination between STS with a simple, translocation-driven karyotype and STS with a complex karyotype harboring primarily copy-number aberrations [83,84]. While MLPS is considered to have a simple karyotype, DDLPS and PLPS represent karyotypically complex STS [83,84,85]. Dancsok et al. observed significantly higher TIL levels in mutation- and/or copy number-driven sarcoma subtypes (including WDLPS and DDLPS) compared to their translocation-associated counterpart (including MLPS) [86]. Thereby, the infiltration rate of translocation-associated sarcoma was closer to that of benign mesenchymal neoplasms. 

Among the LPS subtypes, several reports demonstrated that DDLPS harbors the highest TIL numbers, followed by WDLPS and MLPS [86,87,88,89]. Since PLPS is a rare LPS subtype, data is scarce and inconsistent. Yan et al. observed in PLPS the lowest TIL rate of all subtypes in a cohort of retroperitoneal LPS [88]. In contrast, Oike et al. reported that PLPS have CD8^+^ T cell levels between that of DDLPS and MLPS but exhibit the highest CD4^+^ and FoxP3^+^ levels of all LPS subtypes [89]. 

Interestingly, it has been frequently shown that TIL levels in DDLPS also range among the highest compared to other STS subtypes [86,90,91], which also applies to the percentage of TIL-positive tumors [92]. Moreover, about 40% of DDLPS represent tumors with high T cell infiltration termed immune-hot [93]. Compared to DDLPS, other karyotypically complex STS have a similar or lower proportion of immune-hot tumors. In contrast, studies that lack LPS subtype differentiation reported a relatively low rate of TILs in LPS compared to other STS subtypes [94,95,96,97]. As this observation strongly implies that the heterogeneity of LPS infiltration cannot be reflected by pooling various LPS subtypes, it should be taken into consideration for future study design.

#### 4.1.1. Prognostic Value of T Cells in LPS

Several studies demonstrated that CD8^+^ T cells represent the majority of T cells in LPS, followed by CD4^+^ T cells and FoxP3^+^ Tregs [88,89,98]. However, results regarding the prognostic significance of LPS-infiltrating T cells are less consistent. In a mixed LPS cohort of retroperitoneal origin, survival analysis showed no significant correlation, but high numbers of FoxP3^+^ Tregs tended to predict an unfavorable DFS and OS [88]. 

In the karyotypically complex DDLPS, high numbers of CD4^+^ T cells correlated significantly with a favorable three-year recurrence-free survival (RFS) [98], while another study found no association between CD4^+^, CD8^+^, and FoxP3^+^ infiltration and disease progression [89]. However, a link between FoxP3^+^ Treg infiltration and a prolonged OS was also reported [86]. A more detailed survival analysis based on gene expression data of The Cancer Genome Atlas Sarcoma Collection (TCGA-SARC) dataset revealed that a high Th2 gene signature was significantly associated with a worse DSS in DDLPS [90]. This highlights the necessity of a subset differentiation within the Th compartment, as this may be of prognostic relevance. 

In the karyotypically simple MLPS, Oike et al. observed by using IHC that MLPS patients display significantly lower human leukocyte antigen (HLA) class I expression than DDLPS and PLPS patients suggesting a link to the overall low T cell infiltration [89]. More specifically, about 78% of MLPS samples were negative for HLA class I whereas all DDLPS and PLPS samples exhibited HLA class I expression. CD8^+^ T cell infiltration was positively associated with the level of HLA class I expression both in MLPS and DDLPS. Whether HLA class I is lost or downregulated in MLPS, this finding provides a potential mechanism leading to the overall lowest infiltration rate in MLPS and exposes an obstacle to overcome in terms of T cell-based immunotherapy. Interestingly, a high HLA class I expression in MLPS was associated with an unfavorable PFS, but only 9% of the patients exhibited this feature [89]. A lower, but not absent, HLA class I expression in MLPS compared to WDLPS/DDLPS is further supported by gene expression data [87]. Minopoli et al. reported that T cell infiltration (CD3^+^, CD8^+^, and CD4^+^) was significantly elevated in low-grade compared to high-grade MLPS while infiltration of FoxP3^+^ Tregs was consistently low and independent of grading [99]. In addition, a negative impact of FoxP3^+^ Treg infiltration on the OS of MLPS patients was observed [86]. However, other studies failed to demonstrate an association between CD8^+^, CD4^+^, and FoxP3^+^ T cell levels and PFS in MLPS [89,99].

Studies collectively analyzing T cell infiltration in different STS subtypes (including LPS) via IHC are also inconsistent regarding the prognostic value. While there are reports correlating high CD3^+^, CD8^+^, and CD4^+^ T cell infiltration with a significantly favorable outcome [100,101], others showed that high CD3^+^ and CD4^+^ infiltrates tend to predict a negative prognosis [102]. Furthermore, several studies did not find any association between T cell infiltration and patient outcome [95,96]. The prognostic value of high FoxP3^+^ Treg levels is also undefined in mixed STS cohorts (including LPS) since no impact on OS, but a negative impact on local recurrence in multivariate analysis was demonstrated [95,102]. Besides different examined survival parameters, these conflicting results may be caused by varying cohort characteristics and STS heterogeneity both in terms of genetic alterations and subsequent T cell infiltration as well as clinical features and treatment characteristics. However, contradictory results also occurred within one LPS subtype indicating further mechanisms leading to an inconclusive prognostic value. In this regard, Issels et al. obtained an interesting finding when comparing pre- and post-therapeutic STS samples [103]. While the pre-treatment immune infiltration had no impact on patient outcome, significant correlations with the prognosis were only observed for the post-treatment immune infiltration. The fact that the above-mentioned studies often combine STS with different treatment regimens, both neoadjuvant and adjuvant, implies another reason hampering the definition of the prognostic value of T cells in STS and LPS. In addition, methodological differences regarding both IHC and survival analysis may contribute to inconclusive outcomes.

Several studies used the publicly available TCGA-SARC dataset to investigate a potential association of transcriptomic immune signatures to the clinical outcome. The cohort includes only therapy-naïve, primary tumors of seven different STS histologies and thus allows for improved clinical association due to increased homogeneity [90]. Of note, LPS is only represented by DDLPS which is the only histology in the cohort with 4/50 recurrent cases. Based on these data, Judge et al. demonstrated that a CD8^+^ T cell gene signature was significantly associated with an improved OS, whereas CD4^+^ T cells and Tregs were not related to OS [101]. Zhu and Hou showed that CD8^+^ T cell and Treg gene signatures were significantly associated with prolonged OS [104]. A third study reported no significant correlation between both CD8^+^ T cells and Tregs and OS [105]. Regarding publicly available transcriptome databases, the composition of the patient cohort and, most importantly, the applied gene signatures may vary among the studies and thereby cause conflicting results that are based on the same initial dataset. 

In contrast to a quantitative analysis of the T cell infiltration via IHC or RNA sequencing, an analysis of the T cell receptor (TCR) repertoire provides additional information about the quality of the T cell response and was linked to patient outcomes in different tumor entities [106]. By sequencing the TCR β chain complementary determining region 3, several studies reported low TCR clonality in LPS compared to other tumor entities and other STS subtypes [87,88,94,98]. These findings indicate a diverse intratumoral TCR repertoire that lacks highly expanded T cell clones, which is generally associated with a low specificity of the T cell response [106]. While Pollack et al. observed a similar low TCR clonality for WDLPS/DDLPS and MLPS, MLPS exhibited a lower T cell fraction (reflecting a lower T cell infiltration) compared to WDLPS/DDLPS [87]. Moreover, Schroeder et al. demonstrated in DDLPS that a high TCR clonality combined with a low T cell fraction was linked to significantly worse OS [98]. This implies the need for novel therapeutic approaches that induce an efficient tumor antigen-driven expansion and infiltration of specific effector T cells.

#### 4.1.2. Predictive Value of T Cells in LPS and Their Therapeutic Modulation

In addition to a prognostic significance, the level of T cell infiltration gained interest as a predictive value for the response to immunotherapy-based approaches [107]. Response to pembrolizumab (anti-PD-1 antibody) monotherapy in advanced sarcoma (SARC028 clinical trial) was only seen in DDLPS and undifferentiated pleomorphic sarcoma (UPS) patients [108]. Retrospectively, the response was positively correlated with a higher baseline density of several T cell phenotypes including FoxP3^+^ Tregs and CD45RO^+^ effector memory T cells [15]. In addition, higher FoxP3^+^ Treg percentages and higher CD8^+^ T cell densities prior to immunotherapy were associated with a favorable PFS.

As STS generally exhibit a predominantly low T cell infiltration, termed immune-cold [109], several studies investigated the immunomodulatory properties of conventional anticancer treatments to convert them into immune-hot tumors with high T cell levels [110]. Studies analyzing paired tissue samples via IHC in mixed STS cohorts (including LPS) indeed disclosed therapy-induced priming of T cell infiltration. Sharma et al. reported that around 80% of patients showed elevated expression of HLA class I molecules after radiotherapy and that a majority of patients exhibited higher T cell infiltration (CD3^+^, CD4^+^, and CD8^+^) post-treatment [111]. After chemotherapy with or without regional hyperthermia, increased TIL levels were found in around 40% of STS patients [103]. Focusing solely on LPS, Snow et al. observed that TIL levels before and after radiotherapy remained unchanged for the majority of LPS patients [112]. However, the separation of LPS into the histological subtypes revealed an increase in the TIL score in 64% of all paired DDLPS samples. In contrast, no WDLPS or MLPS patients showed increased TIL scores after radiotherapy. Furthermore, FoxP3^+^ Treg levels were more likely to decrease and the CD8:FoxP3 cell ratio more likely to increase among recurrence-free patients. Once again, these findings underscore the remarkable eligibility of DDLPS for immunotherapy and challenge the suitability of radiotherapy to enhance the accessibility of WDLPS and MLPS for immunotherapy. Yet, given the relatively small cohort size due to the limited availability of paired tissue samples and the fact that different therapeutic regimens were applied in the studies, further analysis of therapeutic modulation is needed. Since MLPS is characterized by an exceptionally low HLA class I expression and showed no increase in TIL levels after radiotherapy [87,89,112], systemic treatment with interferon-γ may be a suitable approach to enhance HLA class I expression and subsequently T cell infiltration, as it was already demonstrated in synovial sarcoma and MLPS patients [113].

### 4.2. B Cells

Studies investigating TILs in STS have traditionally focused on T cells, particularly cytotoxic T cells, due to their outstanding direct antitumor effects and strong correlation with good clinical outcomes. However, recent pioneering studies have shifted the interest to B cells and tertiary lymphoid structures (TLS) [14,114]. As activators of the adaptive humoral immune response, B cells mediate their effector functions via antibody production, cytokine secretion, and antigen-presentation to T cells as professional antigen-presenting cells. These diverse effector mechanisms harbor both antitumor and protumor potential, which was recently reviewed by Sharonov et al. [115]. Thus, the clinical benefit of high B cell, plasma cell, or immunoglobulin levels remains unclear due to contradictory findings within and between tumor entities. Furthermore, B cells are involved in the formation of TLS, which represent ectopic accumulations of lymphocytes resembling the structure of secondary lymphoid organs [116]. Therefore, TLS are sites of lymphocyte proliferation and effector cell differentiation and may play a key role in antitumor immunity. 

#### 4.2.1. Prognostic Value of B Cells in LPS

In LPS as well as mixed STS cohorts (with or without LPS), T cells are generally more frequent than CD20^+^ B cells [88,96,117]. Nevertheless, Sorbye et al. identified high levels of CD20^+^ B cells as an independent positive prognostic marker for DSS in STS (including LPS) with wide resection margins [100]. This was confirmed within the TCGA-SARC cohort (including DDLPS), where high CD20^+^ B cell levels were associated with favorable OS [117]. Combining two independent gene expression databases comprising overall about 500 STS of DDLPS, UPS, and leiomyosarcoma histologies, Petitprez et al. demonstrated that high expression of B cell-associated genes was a strong prognostic marker for an improved OS, independent of CD8^+^ T cell infiltration [14]. However, not all studies investigating CD20^+^ B cells in STS indicate a positive correlation between B cell infiltration and patient survival. While Yan et al. found no significant association with DFS or OS in LPS [88], Smolle et al. reported that CD20^+^ B cells were significantly correlated with an increased risk of recurrence in a mixed STS cohort including LPS [96]. However, this correlation was not reproducible in multivariate analysis. They further analyzed the TCGA-SARC dataset and found no correlation between the expression of B cell-related genes (*CD19*, *MS4A1*, *CD22*, and *CD79A*) and OS using univariate Cox-regression analysis. Thereby, different statistical approaches (stratified vs. unstratified) may have led to these conflicting results within the TCGA-SARC cohort. Smolle et al. further reported that LPS was characterized by the highest B cell infiltration among the studied histological subtypes, confirmed both at protein and RNA levels [96].

Investigating the TME of STS more comprehensively, Petitprez et al. integrated several immune cell populations and malignant cell characteristics to define an immune classification that ranges from the least infiltrated class A to the highly vascularized class C, to class E with the highest expression of immune cell-associated genes [14]. Class D and E STS, both ‘immune-high’, were significantly associated with a prolonged OS compared to other classes. Besides harboring an elevated B cell lineage signature, class E STS exhibited high expression of plasma cell-related genes and CXC-chemokine ligand 13 (CXCL13), known for lymphocyte recruitment to secondary lymphoid organs. Accordingly, they displayed intratumoral TLS in more than 80% of the cases making them a characteristic of class E STS. In addition, TLS-bearing STS were significantly stronger infiltrated by CD3^+^ and CD8^+^ T cells as well as CD20^+^ B cells—even after exclusion of the lymphocytes located inside the TLS. Consistently, Yan et al. reported that retroperitoneal TLS^+^ LPS showed higher TIL proportions and tended to have a favorable DFS and OS [88]. However, Tseng et al. found no significant correlation between TLS-containing retroperitoneal WDLPS/DDLPS and recurrence while the presence of TLS in DDLPS was associated with a shorter OS [118]. However, a small sample size limits the impact of these findings. 

#### 4.2.2. Predictive Value of B Cells in LPS

Beyond the prognostic significance of B cells, the immune classification according to Petitprez et al. had predictive value within the SARC028 study (phase II trial of pembrolizumab in patients with advanced STS). Expectedly, immune class E STS exhibited the highest response rate compared to other classes [14,108]. Following these findings, the PEMBROSARC study (phase II trial of pembrolizumab combined with low-dose cyclophosphamide in patients with advanced STS) was extended by a new patient cohort with TLS^+^ STS of which around one-third represented WDLPS and DDLPS [114]. In contrast, the initial, unselected cohort included just one patient with TLS. The TLS^+^ cohort showed a significantly higher response rate (30% vs. 2%) and survival (median PFS of 4.1 months vs. 1.4 months) in comparison to the unselected cohort. 

Taken together, these findings suggest a major role of intratumoral B cells in STS for both the clinical outcome and response to immunotherapy, potentially mediated by their involvement in TLS. 

### 4.3. Natural Killer (NK) Cells

Belonging to innate immunity, NK cells exhibit a high cytotoxic potential and capacity to secrete a wide range of cytokines. In contrast to CD8^+^ T cells, they do not rely on the presentation of tumor-associated antigens but can instead eliminate cells that avoid T cell recognition by reduced HLA class I expression. Thus, NK cells have a crucial role in cancer immunosurveillance [119] and are associated with increased patient survival across multiple tumor entities [12]. However, due to their plasticity and challenging molecular characterization, studies addressing tumor-infiltrating NK cells are scarce, especially in STS and LPS. 

In a sarcoma cohort of 1072 patients, Dancsok et al. observed that most tissues were completely void of CD56^+^ cells as examined by IHC [86]. However, among all sarcoma subtypes, DDLPS displayed an increased number of tissues that were infiltrated by CD56^+^ cells, although infiltration by CD8^+^, CD4^+^, and FoxP3^+^ T cells was remarkably higher. In contrast, analysis of transcriptomic data of two independent cohorts revealed that the immune infiltration score of NK cells in DDLPS ranges among the scores of CD8^+^ T cells and Th2 cells [90]. Flow cytometry analysis confirmed that in STS (including LPS) CD56^+^ NK cells account for a similar proportion as CD8^+^ T cells of all TILs [101]. 

While the majority of NK cells in the peripheral blood exhibit a CD56^dim^CD16^hi^ phenotype, which is considered primarily cytotoxic, CD56^bright^CD16^-/lo^ NK cells are considered as less cytolytic and rather regulatory [119]. In a mixed STS cohort, of which around 40% represented LPS, no difference in the proportion of CD56^bright^ NK cells between peripheral blood and tumor tissue was observed [101]. For both CD56^bright^ and CD56^dim^ NK cells, the percentage of CD69^+^ NK cells was higher in the tumor tissue compared to peripheral blood with no significant difference between both subsets in the tumor. Nevertheless, intratumoral CD56^dim^ NK cells exhibited significantly elevated levels of the immune checkpoint TIGIT compared to CD56^bright^ NK cells suggesting an impaired functionality. Based on these findings, the authors demonstrated in vitro that combining IL-15 stimulation and TIGIT blockade was able to significantly increase the cytotoxic activity of both NK and T cells, highlighting its potential as a novel treatment strategy for STS and LPS.

#### Prognostic Value of NK Cells in LPS

To our knowledge, at present no study has been published investigating NK cells exclusively in LPS. Therefore, its prognostic value was only assessed in mixed sarcoma cohorts that included LPS. Dancsok et al. observed that a high CD56^+^ score was linked to significantly worse OS in mutation- and/or copy number-driven sarcomas (including WDLPS and DDLPS) while the overall CD56 expression in this cohort was remarkably low [86]. In contrast, a high NK cell-related gene signature was associated with a significantly prolonged OS in the TCGA-SARC cohort (including DDLPS) as confirmed by two independent studies [101,105]. However, a separate analysis of the histological subtypes within the TCGA-SARC cohort revealed that a NK cell signature was not significantly correlated with DSS in DDLPS [90]. Further studies and the implementation of novel NK cell-specific markers or signatures are needed to explore the functional role and prognostic significance of NK cells in the TME of LPS.

### 4.4. Tumor-Associated Macrophages (TAMs)

Within the tumor tissue, macrophages exhibit remarkable plasticity displayed by M1 and M2 macrophages that represent the two extreme cases of a spectrum with several intermediate states. These phenotypes are accompanied by distinct functional profiles with opposing effects, making the role of TAMs in the antitumoral immune response highly complex [120]. Importantly, the polarization is mainly determined by the surrounding micromilieu, shaped by both tumor cells and the TME. Macrophages of M1 polarization are characterized by pro-inflammatory properties and are associated with favorable patient outcomes in several tumor entities [12]. In contrast, M2 macrophages are physiologically involved in wound healing and tissue repair and mediate anti-inflammatory effects and immunosuppression. In line with this, high infiltration with M2 macrophages is overall associated with a poor prognosis [12]. While CD68 is traditionally used as a pan-macrophage marker and CD163 as a marker for M2-like TAMs, novel methods enable a more detailed dissection of their polarization and functional state.

When combining STS subtypes as well as LPS subtypes, macrophages generally constitute the majority of infiltrating immune cells thereby outnumbering TILs, which was confirmed both at protein and RNA levels [89,96,117,121]. Comparing the LPS subtypes, a distribution similar to the T cell infiltration emerges. DDLPS and PLPS exhibit the highest TAM levels, followed by WDLPS and MLPS [89,121]. Moreover, compared to other karyotypically complex STS subtypes, DDLPS exhibits one of the highest TAM infiltrations [90,121]. Interestingly, Dancsok et al. demonstrated that the less infiltrated LPS subtypes WDLPS and MLPS were no longer found to have an excess of TAMs over TILs [121]. In this regard, it was reported that high-grade MLPS are associated with elevated TAM levels compared to low-grade MLPS whereas the opposite applied for TILs, thereby revealing an inverse correlation of TAMs and T cells in MLPS [99]. Since the patient cohort of Dancsok et al. included low- and high-grade tumors and no distinction was made in the analysis, this inverse correlation may be the underlying mechanism of a missing TAM excess in these LPS subtypes [121].

In general, TAMs are considered to be predominantly M2 polarization. Several studies confirmed this for STS, both at protein and RNA levels [117,121]. Moreover, based on gene expression data from the TCGA-SARC cohort, DDLPS exhibited the highest M2:(M0 + M1) ratio among the analyzed STS subtypes [121].

#### 4.4.1. Prognostic Value of TAMs in LPS

Remarkably, TAMs are mainly associated with poor prognosis. Especially in MLPS, a worse patient outcome (PFS and OS) was frequently reported for both high CD68^+^ and CD163^+^ TAM levels [89,99,122] with a significant correlation to the occurrence of metastasis for CD68^+^ cell infiltration [122]. However, the clinical association regarding DDLPS is rather inconclusive. High CD163^+^ TAM infiltration was either observed to have a significantly positive [121] or no significant impact on PFS [89]. A negative association between an M2 macrophage-associated gene signature and three-year survival was also demonstrated [98]. In mixed STS cohorts including LPS, Smolle et al. linked high CD68^+^ TAM levels to a significantly higher risk of local recurrence [96] while Sorbye et al. reported no significant impact of high CD68^+^ TAM levels on DSS [123].

Interestingly, Minopoli et al. reported that primary cancer cells of two high-grade MLPS patients significantly promoted M2 polarization of monocytes in co-culture, and in turn, the monocytes increased the invasiveness and transendothelial migration of MLPS cells, thus providing a potential explanation for the link to poor prognosis [99]. The positive effect of TAMs on motility and invasiveness of MLPS cells in vitro was already demonstrated by a previous study [122]. Additionally, activation of epidermal growth factor receptor (EGFR) in MLPS tumor cells by macrophage-secreted ligand heparin-binding EGF-like growth factor (HB-EGF) plays a crucial role in this process. Accordingly, the presence of phosphorylated EGFR was significantly associated with higher CD68^+^ TAM levels in human MLPS samples, thereby suggesting HB-EGF as a potential drug target in the treatment of MLPS patients [122]. 

#### 4.4.2. Predictive Value of TAMs in LPS and Their Therapeutic Modulation

Apart from cytokines, TAMs mediate immunosuppressive effects via the expression of immune checkpoint molecules. Therefore, TAM infiltration may play a key role in response to CPI therapy. A correlative analysis of immune infiltrates within the SARC028 study (phase II trial of pembrolizumab in patients with advanced STS) reported a significantly higher proportion of PD-L1-expressing CD68^+^ TAMs at baseline among the responders (exclusively UPS and DDLPS) [15]. In addition, PD-L1^+^ TAMs may be a better predictor of anti-PD-1 response than PD-L1 expressing tumor cells since several responders were negative for the latter prior to therapy. In contrast, all responders were positive for PD-L1^+^ TAMs. Interestingly, UPS and DDLPS patients showed a significant decrease in CD68^+^ TAM density in course of the therapy (pre-treatment vs. 8 weeks on-treatment) that was not observed for all STS subtypes together. Combined with a largely unchanged T cell density this suggests that anti-PD-1 treatment may shape the TME towards an effective antitumor landscape in UPS and DDLPS.

However, modulation of TAM infiltration during radiotherapy seems to behave differently. Snow et al. observed that CD68^+^ TAM levels were increased in the majority of LPS patients after radiotherapy, especially in DDLPS [112]. Interestingly, a similar trend towards increased CD163^+^ infiltrates upon radiotherapy was shown in UPS, while the combination with chemotherapy instead led to a slight decrease [124]. Moreover, transcriptomic analysis of a mixed STS cohort, of which one-third represented LPS, indicated that radiotherapy-induced downregulation of molecules and cell markers related to immunosuppression, including interleukin (IL)-10, transforming growth factor (TGF)-β, CD68, and CD163, correlated positively with three-year survival [111]. The fact that anti-PD-1 treatment was able to induce a decrease in TAM infiltration in DDLPS highlights its potential as an effective anticancer treatment. 

**Figure 2:**
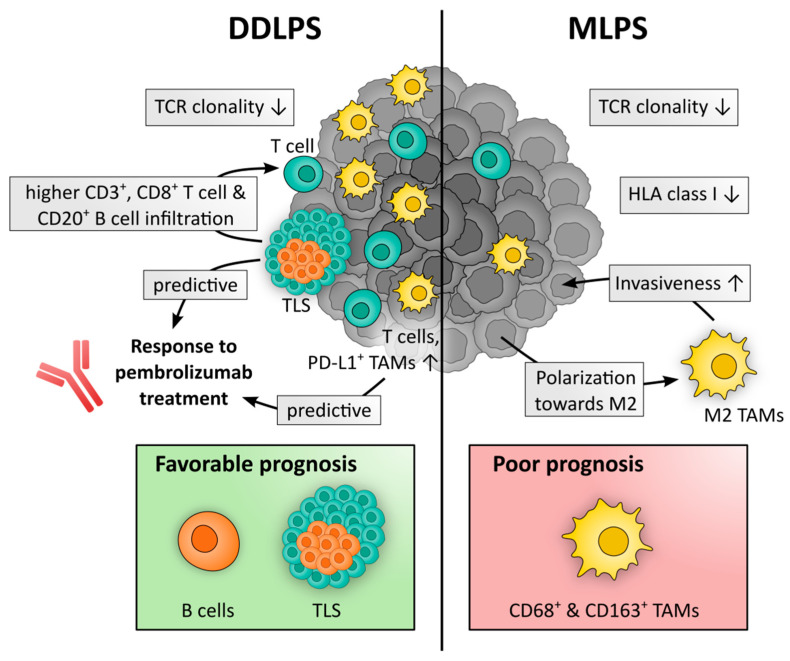
The histological LPS subtypes display different features of their immune contexture. Research mainly focuses on DDLPS and MLPS, whereas insights on the immune microenvironment of WDLPS and PLPS are scarce and lacking in the case of MPLPS. DDLPS is characterized by a higher infiltration of T cells and tumor-associated macrophages (TAMs) compared to MLPS and TAMs are outnumbering T cells in DDLPS while this was not observed in MLPS. Both subtypes exhibit low T cell receptor (TCR) clonality and additionally, MLPS tumors display low levels of human leukocyte antigen (HLA) class I expression. Response to pembrolizumab in DDLPS patients was correlated to a higher density of T cells and a higher proportion of PD-L1^+^ TAMs at baseline. Furthermore, the presence of tertiary lymphoid structures (TLS) in DDLPS is associated with response to pembrolizumab treatment and correlates with elevated infiltration levels of CD3^+^ and CD8^+^ T cells as well as CD20^+^ B cells. In MLPS, tumor cells can promote M2 polarization of TAMs which in turn can enhance the motility and invasiveness of MLPS cells. While several immune cell types are of undefined prognostic value in LPS, accumulating evidence suggests a link to positive prognosis for B cells and TLS in DDLPS and an association with negative prognosis for TAMs, both CD68^+^ and CD163^+^, in MLPS.

A summary of important immune contexture characteristics of DDLPS and MLPS is depicted in Figure 2.

## 5. Soluble and Membrane-Bound Molecules within the TME of LPS and Their Clinical Relevance

Within the constantly evolving microenvironment, non-cellular components are of major importance and significantly affect both the immune response and tumor development, growth, and progression. Among the membrane-bound molecules, immune checkpoints emerged as key molecules harnessed by the tumor to inhibit effective antitumor immunity. Furthermore, cytokines play a crucial role in the TME since they shape various functions of immune cells, which contribute to TME modulation and influence tumor growth. Nevertheless, a comprehensive analysis of the cytokine composition within the TME remains challenging and thus studies addressing the LPS cytokine micromilieu are quite limited. 

### 5.1. Protumoral Soluble Molecules

Due to its effects on tumor progression, metastasis formation, and antitumor immunity, the cytokine IL-6 gained increasing attention and is among the most studied cytokines in the context of LPS. Enhanced IL-6 serum levels in STS (including LPS) patients correlated with higher tumor grade and were a negative prognostic factor for OS, DFS, and event-free survival [125,126]. Furthermore, Rutkowski et al. showed an association between elevated IL-6 serum levels and larger tumor size, and more frequent recurrence [125]. IHC analysis of STS tissues (including WDLPS, DDLPS, and MLPS) confirmed the results obtained from blood serum. High-grade tumors displayed elevated levels of IL-6 and IL-6R compared to low-grade tumors [127]. While Rutkowski et al. did not find any difference in serum levels of IL-6 between patients with and without metastasis [125], simultaneous high expression of both IL-6 and IL-6R was associated with worse OS and metastasis-free survival and thus emerged as prognostic factors [126]. Additionally, upregulated extracellular vesicles containing miR-25-3p and miR-92a-3p were detected in WDLPS/DDLPS patients [128]. Further in vitro analyses including human LPS cell lines and peritoneal murine macrophages showed that miR-25-3p and miR-92a-3p promoted toll-like receptor 7/8–dependent IL-6 secretion in TAMs, which resulted in LPS cell proliferation, migration, and invasion. These observations suggest that serum levels of IL-6 might serve as a feasible, promising prognostic marker, and treatment with monoclonal antibodies targeting IL-6 and/or IL-6R might be of interest for patients with high expression of these molecules [129].

Besides IL-6, various immunosuppressive cytokines have been identified to play significant roles in shaping a protumoral immune landscape and subsequently driving tumor growth. While the clinical significance of the two major cytokines IL-10 and TGF-β has been studied in mixed cohorts of STS, which, to a greater or lesser extent, contained LPS, only a few studies have analyzed them at the level of LPS histological subtype. For example, high expression of the immunosuppressive cytokine TGF-β was associated with shorter DSS in a mixed STS cohort including LPS [123]. Moreover, Mazzu et al. reported that miR-193b directly interacts with a regulator of TGF-β signaling, SMAD4, in WDLPS/DDLPS cells [130]. Interestingly, gene expression of *TGFBR2* was highest in WDLPS/DDLPS in comparison to other STS such as UPS, leiomyosarcoma, or synovial sarcoma [87]. It was further shown by utilizing transcriptomic data from the TCGA-SARC cohort (including DDLPS) that an immunosuppressive profile of the tumor may attenuate the positive prognostic effect of B cells [117]. Importantly, CD20^+^ B cells had no impact on OS in tumors with high IL-10 expression levels, compared to those with low or non-stratified IL-10 expression. This was also demonstrated for high expression levels of the enzyme cyclooxygenase-2 (*PTGS2*). Similar to IL-6, high serum levels of IL-10 were associated with worse DFS and OS in STS patients, including LPS [125]. Moreover, IL-10 was frequently co-expressed with CD163 suggesting M2 TAMs as a potential IL-10 source [117]. Interestingly, high serum levels of macrophage colony-stimulating factor (M-CSF), a critical growth and differentiation factor for macrophages, were correlated to the large tumor size of STS, including LPS [125]. Furthermore, high M-CSF in serum and tumor tissue was linked to a shorter OS in STS patients (including LPS) [123,125]. 

Although further studies are required to comprehensively dissect the LPS cytokine micromilieu, these observations underline the clinical significance and prognostic value of immunosuppressive soluble molecules within the TME of LPS, providing an initial incentive that blocking these pathways may enable novel therapeutic approaches.

### 5.2. Immune Checkpoint Molecules

Numerous immunological checkpoints regulate immune cell activation and function to maintain homeostasis and self-tolerance while preventing autoimmunity. Moreover, they regulate the duration and magnitude of the immune response to prevent excessive damage to healthy tissue. In the context of cancer, long-lasting immune responses and constant high antigen load lead to an upregulation of these molecules and thus a dampening of anti-tumor immune responses [131,132,133]. Immune checkpoint signaling on effector cells then induces loss of proliferation, inhibition of effector functions, or apoptosis. While tumor cells exploit this as an extremely successful immune escape mechanism it was extensively shown that blocking such a pathway holds an immense potential to treat even progressed and metastatic tumors [134]. Thus, various drugs targeting immune checkpoint molecules are being developed and explored. However, the extreme heterogeneity of LPS, both in terms of immunological landscape and clinical behavior, makes the clinical application challenging.

#### 5.2.1. Expression of Immune Checkpoints in LPS

##### PD-1

Due to the significant role of the PD-1/PD-L1 axis in the antitumor immune response, the expression of both has been widely studied in various cancer entities. Wunder et al. demonstrated via IHC that LPS tissues contained very few or no PD-1^+^ cells (92% of LPS had ≤5% of PD-1^+^ cells) [97]. Accordingly, Yan et al. observed that only 23% of retroperitoneal LPS had 1% or higher amounts of PD-1^+^ TILs [88]. While this suggests very low expression of PD-1 within the TME of LPS, another study with 220 LPS tissues showed PD-1 expression in 55% of tumors [135].

When dividing LPS according to histological subtypes, further variations in expression and inconsistencies among studies become evident. Torabi et al. reported that almost all MLPS, WDLPS, and PLPS were PD-1^+^ by IHC, but the mean positive staining decreased in the given order [136]. Other studies showed consistently low expression of PD-1 in MLPS (10%) and higher in WDLPS (around 25%) [86,137]. The highest expression of PD-1 and also the largest differences between studies were reported in DDLPS, where the PD-1 expression varied between 19%, 50%, and 67% [86,92,137]. Notably, Miyake et al. did not show any significant difference in PD-1 expression between WDLPS and DDLPS, UPS or leiomyosarcoma [138], whereas Pollack et al. reported lower PD-1 expression in WDLPS/DDLPS compared to UPS [87]. 

Further flow cytometry analysis revealed that the expression of PD-1 by CD8^+^ T cells in LPS (WDLPS, DDLPS, MLPS) was among the lowest compared to other STS [94]. Specifically, around 50% of LPS-infiltrating CD8^+^ T cells in LPS were positive for PD-1 and about 10% of these also expressed LAG-3. Similarly, Tseng et al. showed that 65% of CD8^+^ T cells expressed PD-1 in retroperitoneal WDLPS/DDLPS [118]. 

##### LAG-3 and TIM-3

LAG-3 and TIM-3 have been widely studied to be used as next-generation targets for immune checkpoint inhibition. Pioneeringly, the first anti-LAG-3 monoclonal antibody has been recently approved by U.S. Food and Drug Administration (FDA) in a combinatorial therapy with nivolumab (anti-PD-1) for the treatment of melanoma patients [139]. In general, the highest proportion of tissues positive for LAG-3 (defined as ≥1 positive cell in any tissue microarray core) was observed in DDLPS (77%), followed by WDLPS (31%) and MLPS (5%) [86]. LAG-3 expression was highly correlated with *CD8A* gene expression in the TCGA-SARC cohort and the predominant expression of LAG-3 by tumor-infiltrating CD8^+^ T cells was then confirmed by IHC [140]. A flow cytometry analysis revealed that approximately 20% of LPS-infiltrating CD8^+^ T cells expressed LAG-3, while no significant difference was observed compared to other STS [94]. 

Similarly, the highest proportion of tissues positive for TIM-3 was found in DDLPS (88%), whereas around half of WDLPS (55%) and just very few MLPS (10%) samples expressed TIM-3 [86]. In a cohort of LPS patients that included WDLPS, DDLPS, and MLPS, only around 3% of tumor-infiltrating CD8^+^ T cells were positive for TIM-3, which was among the lowest compared to other STS [94]. However, MLPS, which was shown to have very low expression of TIM-3 in other studies, accounted for 40% of the cohorts’ patients. 

Interestingly, in the immune classification of STS according to Petitprez et al., class E STS (‘immune- and TLS-high’), and to a lesser extent class D STS (just ‘immune-high’) exhibited high expression of PD-1 and TIM-3 likely due to the general high immune cell signature [14]. However, high levels of LAG-3 were observed only in class E STS.

Altogether, the variation in expression of immune checkpoint molecules across LPS subtypes largely follows the degree of T cell infiltration. Accordingly, DDLPS displays the highest expression of LAG-3 and TIM-3 while a much lower abundance is observed in MLPS tissues. Nevertheless, these observations give further incentive for potential CPI treatment of DDLPS.

##### PD-L1

The expression of PD-L1 by tumor cells is a potent immune evasion mechanism. However, also immune cells, such as dendritic cells, macrophages, and T cells can express PD-L1. Of note, studies investigating PD-L1 expression in STS do not always distinguish between tumor and immune cells. Movva et al. analyzed 220 not further defined LPS tissues and reported PD-L1 positivity (defined as ≥5%) in 77% of samples [135] while 23% of tumors expressed PD-L1 in retroperitoneal LPS [88]. In contrast, another study observed no LPS tissues with tumor or immune PD-L1 expression in ≥5% of cells [97]. Analysis of gene expression data revealed similarities within translocation-associated sarcoma (including MLPS), such as low expression of PD-L1 [141]. Additionally, WDLPS/DDLPS showed a significantly lower PD-L1 score compared to UPS [87]. Further distinction according to histological subtypes disclosed that DDLPS, WDLPS, and MLPS exhibit positivity for PD-L1 at 67%, 50%, and 30%, respectively [137]. However, the number of studied samples was very low (3, 4, and 10, respectively). On the other hand, in a larger cohort including 49 DDLPS patients, PD-L1 positivity was found in only 12% of cases [92]. Surprisingly, Torabi et al. reported only one PD-L1^+^ sample among 64 tissues of WDLPS, MLPS, and PLPS [136], which was further supported by Que et al. who observed not a single PD-L1^+^ sample among 23 LPS tissues [142]. In conclusion, these studies suggest that PD-L1 expression levels, investigated by different methods, vary among histological subtypes of LPS. However, different scoring methods lead to different definitions of PD-L1 positivity, thereby limiting the comparability of the results.

#### 5.2.2. Clinical Significance of Immune Checkpoint Expression in LPS

##### PD-1 and LAG-3

Association of PD-1 expression to clinical parameters revealed that in retroperitoneal LPS the percentage of PD-1^+^ cases was higher in patients exhibiting multiple tumors and that grade 1 LPS displayed higher proportions of PD-1^+^ TILs compared to grade 2 and 3 LPS [88]. However, Miyake et al. failed to show a correlation between PD-1 expression and tumor grading in a mixed cohort of retroperitoneal sarcoma, including LPS [138]. 

In a mixed STS cohort (including LPS), the presence of intratumoral PD-1^+^ cells predicted shorter OS and event-free survival [137]. Similarly, in retroperitoneal sarcoma (including LPS), high levels of PD-1 were associated with shorter RFS [138]. A separate analysis of mutation and/or copy number-driven subtypes (including WDLPS/DDLPS) and translocation-associated subtypes (including MLPS) revealed that high PD-1 expression is linked to shorter OS in the first group, while it did not affect patient prognosis in the translocation-associated group [86]. Conversely, RNA-sequencing revealed that DDLPS patients with an OS of more than three years exhibited higher PD-1 levels [98].

The clinical significance of LAG-3 in LPS remains largely undefined, as to our knowledge only one study showed that high levels of LAG-3 were associated with high tumor grade and shorter OS in a mixed STS cohort [140].

As the above-mentioned studies consisted mainly of mixed STS cohorts and results exclusive to LPS are still rare, comprehensive analyses according to histological subtypes of LPS may reveal new insights. 

##### PD-L1

The percentage of PD-L1^+^ cases in retroperitoneal LPS was higher among patients with multiple tumors and necrosis and was further linked to high tumor grading [88]. In contrast, PD-L1 expression did not correlate with tumor size and tumor grade in DDLPS but tended to associate with metastasis occurrence [92]. Zheng et al. demonstrated that post-recurrent STS (including LPS) exhibited significantly elevated levels of PD-L1^+^ tumor cells and lymphocytes compared to STS at primary diagnosis while the amount of CD8^+^ T cells decreased [143]. 

The intratumoral presence of PD-L1^+^ cells predicted shorter OS and event-free survival in a mixed STS cohort including LPS [137]. In DDLPS, high PD-L1 levels were linked to a significantly shorter five-year OS [92]. Furthermore, Kim et al. reported that the five-year survival rate of PD-1^+^/PD-L1^+^ STS patients was only 13% [137]. In contrast, PD-1^+^/PD-L1^−^ and PD-1^−^/PD-L1^+^ patients as a group as well as PD-1^−^/PD-L1^−^ patients had strongly improved five-year survival rates. 

Moreover, the PD-L1 DNA copy number represents another possible prognostic marker. In the TCGA-SARC cohort, 21% of DDLPS patients displayed PD-L1 copy number gains (CNG) and PD-L1 CNG was also detected in an independent, untreated high-grade STS cohort that included DDLPS [144]. Patients with PD-L1 CNG exhibited higher PD-L1 expression, a significantly higher mutational load, and were associated with shorter OS.

Altogether these results further emphasize the association of the PD-1/PD-L1 axis to poor survival but also encourage the application of drugs blocking this immune checkpoint pathway to foster an efficient antitumor immunity.

## 6. Immunotherapy for LPS

Immunotherapy has changed the treatment algorithms in multiple malignancies and thus, holds considerable promise also for STS patients [145]. Diverse immunotherapeutic approaches are currently being applied with complete responses observed in selected cancer types and individuals [146]. Profound TME analyses have allowed deciphering the immune cell signatures within the tumor and immunotherapies that may target the tumor-infiltrating immune cells are being investigated in clinical trials [147]. To date, 148 clinical trials in patients with LPS have been initiated. Adoptive cell transfer, chimeric antigen receptor (CAR) T cell therapy, cytokine therapy, and CPI treatment remain the major immunotherapies that are subjected to testing in a total of 22 different clinical trials [148]. These trials mainly aim to trigger the antitumor immune responses in patients with these tumors.

### 6.1. Cytokines and Telomerase Vaccines in Clinical Trials

Cytokine administration may not just serve the purpose of non-specific immune stimulation but can also represent a supportive modality to chemotherapy treatment [148]. In LPS, four clinical trials with granulocyte-macrophage colony-stimulating factors, such as filgrastim, pegfilgrastim, and sargramostim, have been completed (NCT00002764, NCT00061984, NCT00025441, NCT00346125). Interestingly, sargramostim was evaluated in a phase I clinical trial for the treatment of LPS in a combination with a 540–548 telomerase vaccine (NCT00069940). Telomerase is considered an attractive immunotherapeutic target as telomerase activity has been described in more than 85% of human cancers [149]. Several attempts to induce peptide-reactive lymphocytes with 540–548 telomerase vaccine were made, however, they failed to demonstrate the immunological efficacy of this approach [150].

### 6.2. CPI in Clinical Trials

CPI therapy has demonstrated encouraging therapeutic outcomes with a relatively good safety profile which gave a rationale for the addition of CPI to other modalities [151].

It has been previously shown that as compared to other STS subtypes, DDLPS has a relatively high T cell infiltration, together with a higher PD-1 expression [86,90,91]. For that reason, DDLPS has become the predominant LPS subtype evaluated in clinical trials with CPI. MLPS, on the other hand, was associated with the lowest T cell infiltration among LPS suggesting a rather limited efficacy of CPI therapy [87,89]. Clinical trials evaluating the efficacy and safety of CPI therapy in participants with either PLPS or MPLPS are currently missing.

In a study by D’Angelo et al., nivolumab and ipilimumab were evaluated in patients with STS, including those with locally advanced LPS and unresectable LPS [152]. In this multi-center phase II study, forty-three patients were randomly assigned to nivolumab treatment alone and forty-two patients to concomitant treatment with nivolumab and ipilimumab (NCT02500797). In the monotherapy arm, the clinical benefit rate at 12 months was only 2%. Nivolumab and ipilimumab combination showed encouraging objective response rates, however, only in selected histological subtypes. Patients with LPS did not display clinically relevant responses which may be due to their low expression of genes related to T cell infiltration [87,152].

Another phase II clinical trial is currently evaluating nivolumab with and without ipilimumab and radiation therapy in a neoadjuvant setting for the treatment of both resectable and recurrent DDLPS (NCT03307616). In this randomized open-label clinical trial, RFS and OS will be assessed at 12 and 24 months. Similarly, another study of nivolumab in combination therapy has been initiated as an open-label phase I clinical trial of nivolumab and intratumoral BO-112, a nanoplexed form of polyinosinic:polycytidylic acid (poly I:C) for patients with resectable DDLPS. The trial is currently recruiting patients (NCT04420975). Since BO-112 was previously shown to induce local and systemic immunotherapeutic effects by increasing tumor cell apoptosis and enhancing immune reactivity, the primary objective of this study is to determine the safety and tolerability of adding BO-112 to nivolumab treatment (NCT04420975) [153]. 

Among other clinical trials with CPI, a study evaluating the efficacy and safety of anti-PD-1 therapy together with a conditionally active biologic (CAB) AXL-targeted antibody-drug conjugate (CAB-AXL-ADC) has reached phase II of clinical testing and is currently recruiting LPS patients (NCT03425279). 

Avelumab, an anti-PD-L1 antibody, was subjected to testing in phase I/II open-label clinical trials together with chemotherapy in patients with leiomyosarcoma and LPS [154]. A partial response was observed in 13% of the study participants and 43% of the patients had stable disease (SD) as the best response [154]. However, the trial did not meet the primary objective response rate endpoint and was terminated in 2020 (NCT03074318). 

Despite these rather disappointing results in LPS patients, avelumab still represents a promising agent that is being evaluated mostly in combination therapies. A recruiting phase 1 clinical trial aims to determine the safest dose of DCC-3014, a tyrosine kinase inhibitor, that can be administered together with avelumab to participants with advanced or metastatic sarcomas, including those with DDLPS. DCC-3014 is a highly specific inhibitor of the receptor of the colony-stimulating factor-1 (CSF-1R) that has shown promising results in the treatment of rare tenosynovial giant cell tumors [155]. The efficacy and safety of DCC-3014 and avelumab combination in DDLPS remains to be determined. 

Atezolizumab, another anti-PD-L1 agent, has been tested in LPS patients together with prime-boost immunotherapy targeting NY-ESO-1 called CMB305 [156]. Although the combination of atezolizumab and CMB305 did not significantly improve the OS of the study participants, the dual administration led to an induction of NY-ESO-1-specific T cells and promoted antibody responses [156].

Compelling results in patients with LPS were observed in a multicenter open-label phase II clinical trial of pembrolizumab monotherapy (SARC028) in 80 patients with bone and soft tissue sarcomas. Objective responses were observed in 18% of the STS patients, with responses seen only in LPS and UPS [108]. However, one of the most interesting findings of this study was that in the LPS tumors, clinical responses were observed despite the absence of PD-L1 expression [108].

Pembrolizumab is further being applied in another open-label clinical trial with 57 participants diagnosed with STS, including LPS. The trial is currently evaluating the efficacy and safety of pembrolizumab and eribulin in combination treatment (NCT03899805). Eribulin, a nontaxane microtubule inhibitor, has been approved by the FDA for the treatment of patients with LPS who have received a prior anthracycline-containing regimen [157]. In this trial, chemotherapy treatment with eribulin is expected to increase the response to immunotherapy with pembrolizumab. The estimated completion is in August 2024.

### 6.3. Adoptive Transfer, CAR T Cells, and Oncolytic Viruses in Clinical Trials

NY-ESO-1 and MAGE-A4 were identified as important target antigens for the treatment of patients with MLPS [158,159]. Both antigens were shown to be of strong diagnostic value and immunotherapy targeting NY-ESO-1 and MAGE-A4 in MLPS was supported by multiple studies [159]. To date, a single phase I clinical trial has been initiated in patients who have the appropriate HLA-A2 tissue marker and whose LPS tumor has the MAGE-A4 protein expression. In this study, the tolerability and safety of autologous genetically modified MAGE-A4ᶜ¹º³² T cells will be determined (NCT03132922).

In a recent study, MLPS was shown to express high levels of NY-ESO-1 in up to 70% of the cases [160]. Since NY-ESO-1 has been already successfully targeted with adoptive cell therapy, another noticeable clinical trial utilizes autologous NY-ESO-1-specific CD8^+^ T lymphocytes, chemotherapy, and IL-2 with or without dendritic cell-targeting lentiviral vector ID-LV305 (LV305) in the treatment for MLPS. The trial is currently an active open-label phase I clinical trial (NCT03450122) with a total of 15 study participants. The safety of adoptively transferred CD8^+^ T cells targeting NY-ESO-1 positive tumors will be assessed in both the monotherapy setting and in the combination with antigen-specific vaccination. With one of the primary objectives being the assessment of in vivo persistence of NY-ESO-1-specific CD8^+^ T cells, the results of this trial are eagerly awaited in December 2022. 

Several other phase I clinical trials have been initiated in patients with LPS, including a first-in-human multicentre study (NCT05120271) of CAR T cells engineered to express glypican-3 (GPC3) CARs. The GPC3-CAR T cells administration is scheduled in patients with myxoid/round cell LPS after a lymphodepleting chemotherapy and the primary outcome measures aim to assess the safety of such an approach. GPC3-CAR T cells were previously shown to efficiently eradicate liver cancer in vivo and thus, also bring a great deal of promise in LPS [161]. 

Another CAR T cell study with GPC3-CAR T cells is scheduled to start in July 2023 (NCT04715191). In this study, GPC3-CAR T cells will be administered to patients with GPC3-positive LPS together with IL-15 and IL-21 along with lymphodepleting chemotherapy, cytoxan, and fludarabine. Similarly, a phase I clinical trial with GPC3-CAR T cells and IL-15 (AGAR T cells) is currently recruiting patients with the diagnosis of LPS (NCT04377932).

Oncolytic viral immunotherapy with Talimogene laherparepvec (T-VEC) has entered phase II of clinical evaluation in STS, including LPS. In this study, the administration of T-VEC is designed for combination with radiation therapy in localized tumors during neoadjuvant treatment (NCT02923778).

## 7. Conclusions

A growing body of literature investigates the immune contexture of STS and LPS, thereby confirming that its dissection is a highly relevant topic in light of recent advances in immunotherapy. Accumulating evidence emphasizes that there are major differences in the immune landscape of the histological LPS subtypes that may not only have implications for clinical behavior but also their eligibility for novel immunotherapy. In particular, it emerges that MLPS has a largely quiescent immune microenvironment with several potential immunosuppressive mechanisms such as low HLA class I expression and promotion of M2 polarization of TAMs. In contrast, DDLPS displays a highly infiltrated microenvironment, even in comparison to other STS subtypes. Concomitantly, the highest levels of immune checkpoints, e.g., PD-1, LAG-3, and TIM-3 were observed in DDLPS compared to the other LPS subtypes. Unique characteristics are also reflected in the prognostic significance of immune cell infiltrates as B cells emerged as a strong prognostic factor for prolonged survival in DDLPS, mainly driven by analysis of the TCGA-SARC cohort including DDLPS as the only LPS subtype. In contrast, accumulating evidence suggests TAMs as a marker for poor prognosis in MLPS, whereas such consistent association was not observed in DDLPS. However, data for WDLPS and PLPS is scarce, and studies investigating MPLPS are entirely lacking, impeding a detailed characterization of their immune contexture. 

So far, the success of immunotherapeutic strategies in LPS is limited. Therefore, a thorough characterization of LPS-infiltrating immune cells as well as the immunogenicity of tumor cells is crucial to overcome these challenges. On the one hand, it guides the development of selection strategies for the most responsive patient cohort. Since TLS have proven a strong predictive value for response to CPI therapy in STS including LPS, tailored patient cohorts based on this biomarker may dramatically broaden the benefit of CPI therapy in LPS. On the other hand, it drives the identification of prospective immunotherapeutic targets specifically for LPS. Delivering a “don’t eat me” signal, expression of CD47 on tumor cells was demonstrated to be among the highest in DDLPS and PLPS compared to other sarcoma subtypes [121]. Moreover, expression of the corresponding ligand SIRP-α on macrophages was most frequent in DDLPS, followed by WDLPS and MLPS compared to other sarcomas. Equally encouraging are expression levels of the costimulatory molecule OX40 in DDLPS, as DDLPS exhibited the second highest OX40 score of various tumor entities within the TCGA dataset [93]. These findings suggest that LPS and particularly DDLPS are worthy candidates for treatment strategies targeting the CD47-SIRP-α or OX40-OX40L signaling pathways. A multitude of clinical trials investigates the therapeutic manipulation of these axes, however, none of these trials focuses on LPS. Besides targeting specific immune cells or molecules, treatment modalities that augment tumor-specific immunity and induce T cell recruitment may broaden the use of CPI treatment in LPS. As such, radiation therapy or chemotherapy prior to immunotherapy is evaluated by several ongoing clinical trials in STS. However, only a limited number of studies investigated therapy-induced modulation of LPS-infiltrating immune cells. Further characterization of these changes may lead to an improved design of combinatorial treatment strategies in LPS.

However, a major limitation of several studies presented is an insufficient distinction between STS and LPS subtypes. Therefore, a detailed deciphering of the LPS immune contexture requires a broad awareness that grouping of different LPS subtypes does not pay respect to their unique characteristics. This is particularly crucial as dissection of the immune architecture displays a key driver for the design and improvement of efficient immunotherapeutic strategies to combat LPS.

## References

[1] Bourcier K., le Cesne A., Tselikas L., Adam J., Mir O., Honore C., de Baere T. (2019). Basic Knowledge in Soft Tissue Sarcoma. Cardiovasc. Interv. Radiol..

[2] Jones R.L., Lee A.T.J., Thway K., Huang P.H. (2018). Clinical and Molecular Spectrum of Liposarcoma. J. Clin. Oncol..

[3] Saponara M., Stacchiotti S., Gronchi A. (2017). Pharmacological Therapies for Liposarcoma. Expert Rev. Clin. Pharmacol..

[4] Suarez-Kelly L.P., Baldi G.G., Gronchi A. (2019). Pharmacotherapy for Liposarcoma: Current State of the Art and Emerging Systemic Treatments. Expert Opin. Pharmacother..

[5] Abbas Manji G., Singer S., Koff A., Schwartz G.K. (2015). Application of Molecular Biology to Individualize Therapy for Patients with Liposarcoma. Am. Soc. Clin. Oncol. Educ. Book.

[6] Creytens D., Folpe A.L., Koelsche C., Mentzel T., Ferdinande L., van Gorp J.M., van der Linden M., Raman L., Menten B., Fritchie K. (2021). Myxoid Pleomorphic Liposarcoma-a Clinicopathologic, Immunohistochemical, Molecular Genetic and Epigenetic Study of 12 Cases, Suggesting a Possible Relationship with Conventional Pleomorphic Liposarcoma. Mod. Pathol..

[7] Choi J.H., Ro J.Y. (2021). The 2020 WHO Classification of Tumors of Soft Tissue: Selected Changes and New Entities. Adv. Anat. Pathol..

[8] Haddox C.L., Riedel R.F. (2021). Recent Advances in the Understanding and Management of Liposarcoma. Fac. Rev..

[9] Lu J., Wood D., Ingley E., Koks S., Wong D. (2021). Update on Genomic and Molecular Landscapes of Well-Differentiated Liposarcoma and Dedifferentiated Liposarcoma. Mol. Biol. Rep..

[10] Blay J.Y., Honoré C., Stoeckle E., Meeus P., Jafari M., Gouin F., Anract P., Ferron G., Rochwerger A., Ropars M. (2019). Surgery in Reference Centers Improves Survival of Sarcoma Patients: A Nationwide Study. Ann. Oncol..

[11] Manji G.A., Schwartz G.K. (2016). Managing Liposarcomas: Cutting Through the Fat. J. Oncol. Pract..

[12] Bruni D., Angell H.K., Galon J. (2020). The Immune Contexture and Immunoscore in Cancer Prognosis and Therapeutic Efficacy. Nat. Rev. Cancer.

[13] Helmink B.A., Reddy S.M., Gao J., Zhang S., Basar R., Thakur R., Yizhak K., Sade-Feldman M., Blando J., Han G. (2020). B Cells and Tertiary Lymphoid Structures Promote Immunotherapy Response. Nature.

[14] Petitprez F., de Reyniès A., Keung E.Z., Chen T.W.W., Sun C.M., Calderaro J., Jeng Y.M., Hsiao L.P., Lacroix L., Bougoüin A. (2020). B Cells Are Associated with Survival and Immunotherapy Response in Sarcoma. Nature.

[15] Keung E.Z., Burgess M., Salazar R., Parra E.R., Rodrigues-Canales J., Bolejack V., van Tine B.A., Schuetze S.M., Attia S., Riedel R.F. (2020). Correlative Analyses of the SARC028 Trial Reveal an Association between Sarcoma-Associated Immune Infiltrate and Response to Pembrolizumab. Clin. Cancer Res..

[16] Coindre J.M., Pédeutour F., Aurias A. (2010). Well-Differentiated and Dedifferentiated Liposarcomas. Virchows Arch..

[17] Lin O., Zakowski M.F. (2008). Cytology of Soft Tissue, Bone, and Skin. Comprehensive Cytopathology.

[18] Thway K. (2019). Well-Differentiated Liposarcoma and Dedifferentiated Liposarcoma: An Updated Review. Semin. Diagn. Pathol..

[19] Moulin B., Messiou C., Crombe A., Kind M., Hohenberger P., Rutkowski P., van Houdt W.J., Strauss D., Gronchi A., Bonvalot S. (2022). Diagnosis Strategy of Adipocytic Soft-Tissue Tumors in Adults: A Consensus from European Experts. Eur. J. Surg. Oncol..

[20] Fabbroni C., Fucà G., Ligorio F., Fumagalli E., Barisella M., Collini P., Morosi C., Gronchi A., Tos A.P.D., Casali P.G. (2021). Impact of Pathological Stratification on the Clinical Outcomes of Advanced Well-Differentiated/Dedifferentiated Liposarcoma Treated with Trabectedin. Cancers.

[21] de Vita A., Mercatali L., Recine F., Pieri F., Riva N., Bongiovanni A., Liverani C., Spadazzi C., Miserocchi G., Amadori D. (2016). Current Classification, Treatment Options, and New Perspectives in the Management of Adipocytic Sarcomas. Onco Targets Ther..

[22] Kammerer-Jacquet S.F., Thierry S., Cabillic F., Lannes M., Burtin F., Henno S., Dugay F., Bouzillé G., Rioux-Leclercq N., Belaud-Rotureau M.A. (2017). Differential Diagnosis of Atypical Lipomatous Tumor/Well-Differentiated Liposarcoma and Dedifferentiated Liposarcoma: Utility of P16 in Combination with MDM2 and CDK4 Immunohistochemistry. Hum. Pathol..

[23] Bill K.L.J., Seligson N.D., Hays J.L., Awasthi A., Demoret B., Stets C.W., Duggan M.C., Bupathi M., Brock G.N., Millis S.Z. (2019). Degree of MDM2 Amplification Affects Clinical Outcomes in Dedifferentiated Liposarcoma. Oncologist.

[24] Doyle L.A. (2014). Surgical Pathology of Sarcomas. Pathobiology of Human Disease: A Dynamic Encyclopedia of Disease Mechanisms.

[25] Tyler R., Wanigasooriya K., Taniere P., Almond M., Ford S., Desai A., Beggs A. (2020). A Review of Retroperitoneal Liposarcoma Genomics. Cancer Treat. Rev..

[26] Binh M.B.N., Sastre-Garau X., Guillou L., de Pinieux G., Terrier P., Lagacé R., Aurias A., Hostein I., Coindre J.M. (2005). MDM2 and CDK4 Immunostainings Are Useful Adjuncts in Diagnosing Well-Differentiated and Dedifferentiated Liposarcoma Subtypes: A Comparative Analysis of 559 Soft Tissue Neoplasms with Genetic Data. Am. J. Surg. Pathol..

[27] Montella L., Altucci L., Sarno F., Buonerba C., de Simone S., Facchini B.A., Franzese E., de Vita F., Tafuto S., Berretta M. (2021). Toward a Personalized Therapy in Soft-Tissue Sarcomas: State of the Art and Future Directions. Cancers.

[28] Zhang K., Chu K., Wu X., Gao H., Wang J., Yuan Y.C., Loera S., Ho K., Wang Y., Chow W. (2013). Amplification of FRS2 and Activation of FGFR/FRS2 Signaling Pathway in High-Grade Liposarcoma. Cancer Res..

[29] Dehner C.A., Hagemann I.S., Chrisinger J.S.A. (2021). Retroperitoneal Dedifferentiated Liposarcoma. Am. J. Clin. Pathol..

[30] Dang T.N., Tiongco R.P., Brown L.M., Taylor J.L., Lyons J.M., Lau F.H., Floyd Z.E. (2022). Expression of the Preadipocyte Marker ZFP423 Is Dysregulated between Well-Differentiated and Dedifferentiated Liposarcoma. BMC Cancer.

[31] Kim Y.J., Yu D.B., Kim M., Choi Y.L. (2019). Adipogenesis Induces Growth Inhibition of Dedifferentiated Liposarcoma. Cancer Sci..

[32] Murphey M.D., Arcara L.K., Fanburg-Smith J. (2005). From the Archives of the AFIP: Imaging of Musculoskeletal Liposarcoma with Radiologic-Pathologic Correlation. Radiographics.

[33] Danieli M., Swallow C.J., Gronchi A. (2022). How to Treat Liposarcomas Located in Retroperitoneum. Eur. J. Surg. Oncol..

[34] Wen Y., He X., Zhao M. (2021). Dedifferentiated Liposarcoma with Abrupt Transition of Low-Grade and High-Grade Dedifferentiation: A Rare Case Report. Int. J. Immunopathol. Pharmacol..

[35] Tseng W.W., Barretta F., Baia M., Barisella M., Radaelli S., Callegaro D., Yoon D.H., Fiore M., Gronchi A. (2021). Dedifferentiation within Well-Differentiated Liposarcoma of the Extremity or Trunk: Implications for Clinical Management. J. Surg. Oncol..

[36] Beird H.C., Wu C.C., Ingram D.R., Wang W.L., Alimohamed A., Gumbs C., Little L., Song X., Feig B.W., Roland C.L. (2018). Genomic Profiling of Dedifferentiated Liposarcoma Compared to Matched Well-Differentiated Liposarcoma Reveals Higher Genomic Complexity and a Common Origin. Cold Spring Harb. Mol. Case Stud..

[37] Barretina J., Taylor B.S., Banerji S., Ramos A.H., Lagos-Quintana M., Decarolis P.L., Shah K., Socci N.D., Weir B.A., Ho A. (2010). Subtype-Specific Genomic Alterations Define New Targets for Soft-Tissue Sarcoma Therapy. Nat. Genet..

[38] Crago A.M., Socci N.D., DeCarolis P., O’Connor R., Taylor B.S., Qin L.X., Antonescu C.R., Singer S. (2012). Copy Number Losses Define Subgroups of Dedifferentiated Liposarcoma with Poor Prognosis and Genomic Instability. Clin. Cancer Res..

[39] Thway K., Jones R.L., Noujaim J., Zaidi S., Miah A.B., Fisher C. (2016). Dedifferentiated Liposarcoma: Updates on Morphology, Genetics, and Therapeutic Strategies. Adv. Anat. Pathol..

[40] Tap W.D., Eilber F.C., Ginther C., Dry S.M., Reese N., Barzan-Smith K., Chen H.-W., Wu H., Eilber F.R., Slamon D.J. (2011). Evaluation of Well-Differentiated/de-Differentiated Liposarcomas by High-Resolution Oligonucleotide Array-Based Comparative Genomic Hybridization. Genes Chromosomes Cancer.

[41] Takahira T., Oda Y., Tamiya S., Yamamoto H., Kobayashi C., Izumi T., Ito K., Iwamoto Y., Tsuneyoshi M. (2005). Alterations of the RB1 Gene in Dedifferentiated Liposarcoma. Mod. Pathol..

[42] Saifuddin A., Andrei V., Rajakulasingam R., Oliveira I., Seddon B. (2021). Magnetic Resonance Imaging of Trunk and Extremity Myxoid Liposarcoma: Diagnosis, Staging, and Response to Treatment. Skelet. Radiol..

[43] Tariq H., Sarfraz T., Saeed I. (2020). Myxoid Liposarcoma with Cartilagenous Differentiation. J. Coll. Physicians Surg. Pak..

[44] Mujtaba B., Wang F., Taher A., Aslam R., Madewell J.E., Nassar S. (2021). Myxoid Liposarcoma With Skeletal Metastases: Pathophysiology and Imaging Characteristics. Curr. Probl. Diagn. Radiol..

[45] Codenotti S., Mansoury W., Pinardi L., Monti E., Marampon F., Fanzani A. (2019). Animal Models of Well-Differentiated/Dedifferentiated Liposarcoma: Utility and Limitations. Onco Targets Ther..

[46] Yu J.S.E., Colborne S., Hughes C.S., Morin G.B., Nielsen T.O. (2019). The FUS-DDIT3 Interactome in Myxoid Liposarcoma. Neoplasia.

[47] Scapa J.V., Cloutier J.M., Raghavan S.S., Peters-Schulze G., Varma S., Charville G.W. (2021). DDIT3 Immunohistochemistry Is a Useful Tool for the Diagnosis of Myxoid Liposarcoma. Am. J. Surg. Pathol..

[48] Zhu G., Benayed R., Ho C., Mullaney K., Sukhadia P., Rios K., Berry R., Rubin B.P., Nafa K., Wang L. (2019). Diagnosis of Known Sarcoma Fusions and Novel Fusion Partners by Targeted RNA Sequencing with Identification of a Recurrent ACTB-FOSB Fusion in Pseudomyogenic Hemangioendothelioma. Mod. Pathol..

[49] Anderson W.J., Jo V.Y. (2019). Pleomorphic Liposarcoma: Updates and Current Differential Diagnosis. Semin. Diagn. Pathol..

[50] Wan L., Tu C., Qi L., Li Z. (2021). Survivorship and Prognostic Factors for Pleomorphic Liposarcoma: A Population-Based Study. J. Orthop. Surg. Res..

[51] Downes K.A., Goldblum J.R., Montgomery E.A., Fisher C. (2001). Pleomorphic Liposarcoma: A Clinicopathologic Analysis of 19 Cases. Mod. Pathol..

[52] Dermawan J.K., Hwang S., Wexler L., Tap W.D., Singer S., Vanderbilt C.M., Antonescu C.R. (2022). Myxoid Pleomorphic Liposarcoma Is Distinguished from Other Liposarcomas by Widespread Loss of Heterozygosity and Significantly Worse Overall Survival: A Genomic and Clinicopathologic Study. Mod. Pathol..

[53] Gami S., Tiwari S.B., Gautam K., Sharma S., Shrivastav S., Sapkota R. (2021). A Rare Case of Myxoid Pleomorphic Liposarcoma in an Infant: A Report. Int. J. Surg. Case Rep..

[54] Alaggio R., Coffin C.M., Weiss S.W., Bridge J.A., Issakov J., Oliveira A.M., Folpe A.L. (2009). Liposarcomas in Young Patients: A Study of 82 Cases Occurring in Patients Younger than 22 Years of Age. Am. J. Surg. Pathol..

[55] Zare S.Y., Leivo M., Fadare O. (2020). Recurrent Pleomorphic Myxoid Liposarcoma in a Patient With Li-Fraumeni Syndrome. Int. J. Surg. Pathol..

[56] Rizer M., Singer A.D., Edgar M., Jose J., Subhawong T.K. (2016). The Histological Variants of Liposarcoma: Predictive MRI Findings with Prognostic Implications, Management, Follow-up, and Differential Diagnosis. Skelet. Radiol..

[57] Mansfield S.A., Pollock R.E., Grignol V.P. (2018). Surgery for Abdominal Well-Differentiated Liposarcoma. Curr. Treat. Options Oncol..

[58] Kito M., Yoshimura Y., Isobe K., Aoki K., Momose T., Suzuki S., Tanaka A., Sano K., Akahane T., Kato H. (2015). Clinical Outcome of Deep-Seated Atypical Lipomatous Tumor of the Extremities with Median-Term Follow-up Study. Eur. J. Surg. Oncol..

[59] von Mehren M., Randall R.L., Benjamin R.S., Boles S., Bui M.M., Ganjoo K.N., George S., Gonzalez R.J., Heslin M.J., Kane J.M. (2018). Soft Tissue Sarcoma, Version 2.2018: Clinical Practice Guidelines in Oncology. J. Natl. Compr. Cancer Netw..

[60] Crago A.M., Dickson M.A. (2016). Liposarcoma: Multimodality Management and Future Targeted Therapies. Surg. Oncol. Clin. N. Am..

[61] Zagars G.K., Ballo M.T., Pisters P.W.T., Pollock R.E., Patel S.R., Benjamin R.S. (2003). Preoperative vs. Postoperative Radiation Therapy for Soft Tissue Sarcoma: A Retrospective Comparative Evaluation of Disease Outcome. Int. J. Radiat. Oncol. Biol. Phys..

[62] le Cesne A., Ouali M., Leahy M.G., Santoro A., Hoekstra H.J., Hohenberger P., van Coevorden F., Rutkowski P., van Hoesel R., Verweij J. (2014). Doxorubicin-Based Adjuvant Chemotherapy in Soft Tissue Sarcoma: Pooled Analysis of Two STBSG-EORTC Phase III Clinical Trials. Ann. Oncol..

[63] Gronchi A., Ferrari S., Quagliuolo V., Broto J.M., Pousa A.L., Grignani G., Basso U., Blay J.Y., Tendero O., Beveridge R.D. (2017). Histotype-Tailored Neoadjuvant Chemotherapy versus Standard Chemotherapy in Patients with High-Risk Soft-Tissue Sarcomas (ISG-STS 1001): An International, Open-Label, Randomised, Controlled, Phase 3, Multicentre Trial. Lancet Oncol..

[64] Eilber F.C., Eilber F.R., Eckardt J., Rosen G., Riedel E., Maki R.G., Brennan M.F., Singer S. (2004). The Impact of Chemotherapy on the Survival of Patients with High-Grade Primary Extremity Liposarcoma. Ann. Surg..

[65] Gahvari Z., Parkes A. (2020). Dedifferentiated Liposarcoma: Systemic Therapy Options. Curr. Treat. Options Oncol..

[66] Bonvalot S., Gronchi A., le Péchoux C., Swallow C.J., Strauss D., Meeus P., van Coevorden F., Stoldt S., Stoeckle E., Rutkowski P. (2020). Preoperative Radiotherapy plus Surgery versus Surgery Alone for Patients with Primary Retroperitoneal Sarcoma (EORTC-62092: STRASS): A Multicentre, Open-Label, Randomised, Phase 3 Trial. Lancet Oncol..

[67] Callegaro D., Raut C.P., Ajayi T., Strauss D., Bonvalot S., Ng D., Stoeckle E., Fairweather M., Rutkowski P., van Houdt W.J. (2022). Preoperative Radiotherapy in Patients with Primary Retroperitoneal Sarcoma: EORTC-62092 Trial (STRASS) Versus Off-Trial (STREXIT) Results. Ann. Surg..

[68] Dürr H.R., Rauh J., Baur-Melnyk A., Knösel T., Lindner L., Roeder F., Jansson V., Klein A. (2018). Myxoid Liposarcoma: Local Relapse and Metastatic Pattern in 43 Patients. BMC Cancer.

[69] Visgauss J.D., Wilson D.A., Perrin D.L., Colglazier R., French R., Mattei J.C., Griffin A.M., Wunder J.S., Ferguson P.C. (2021). Staging and Surveillance of Myxoid Liposarcoma: Follow-up Assessment and the Metastatic Pattern of 169 Patients Suggests Inadequacy of Current Practice Standards. Ann. Surg. Oncol..

[70] Patel S.R., Andrew Burgess M., Plager C., Papadopoulos N.E., Linke K.A., Benjamin R.S. (1994). Myxoid Liposarcoma. Experience with Chemotherapy. Cancer.

[71] Pitson G., Robinson P., Wilke D., Kandel R.A., White L., Griffin A.M., Bell R.S., Catton C.N., Wunder J.S., O’Sullivan B. (2004). Radiation Response: An Additional Unique Signature of Myxoid Liposarcoma. Int. J. Radiat. Oncol. Biol. Phys..

[72] Issels R.D., Lindner L.H., Verweij J., Wessalowski R., Reichardt P., Wust P., Ghadjar P., Hohenberger P., Angele M., Salat C. (2018). Effect of Neoadjuvant Chemotherapy plus Regional Hyperthermia on Long-Term Outcomes among Patients with Localized High-Risk Soft Tissue Sarcoma the EORTC 62961-ESHO 95 Randomized Clinical Trial. JAMA Oncol..

[73] Oei A.L., Kok H.P., Oei S.B., Horsman M.R., Stalpers L.J.A., Franken N.A.P., Crezee J. (2020). Molecular and Biological Rationale of Hyperthermia as Radio- and Chemosensitizer. Adv. Drug Deliv. Rev..

[74] Lee S., Son B., Park G., Kim H., Kang H., Jeon J., Youn H., Youn B. (2018). Immunogenic Effect of Hyperthermia on Enhancing Radiotherapeutic Efficacy. Int. J. Mol. Sci..

[75] Neuwirth M.G., Song Y., Sinnamon A.J., Fraker D.L., Zager J.S., Karakousis G.C. (2017). Isolated Limb Perfusion and Infusion for Extremity Soft Tissue Sarcoma: A Contemporary Systematic Review and Meta-Analysis. Ann. Surg. Oncol..

[76] Judson I., Verweij J., Gelderblom H., Hartmann J.T., Schöffski P., Blay J.Y., Kerst J.M., Sufliarsky J., Whelan J., Hohenberger P. (2014). Doxorubicin Alone versus Intensified Doxorubicin plus Ifosfamide for First-Line Treatment of Advanced or Metastatic Soft-Tissue Sarcoma: A Randomised Controlled Phase 3 Trial. Lancet Oncol..

[77] Zijoo R., von Mehren M. (2016). Efficacy of Trabectedin for the Treatment of Liposarcoma. Expert Opin. Pharmacother..

[78] Lee A.T.J., Jones R.L., Huang P.H. (2019). Pazopanib in Advanced Soft Tissue Sarcomas. Signal Transduct. Target. Ther..

[79] Assi T., Kattan J., el Rassy E., Honore C., Dumont S., Mir O., le Cesne A. (2019). A Comprehensive Review of the Current Evidence for Trabectedin in Advanced Myxoid Liposarcoma. Cancer Treat. Rev..

[80] Sobczuk P., Bątruk H., Wójcik P., Iwaniak K., Kozak K., Rutkowski P. (2022). In Search of Effective Therapies: The Current Landscape of Phase II Trials in Patients with Advanced Soft Tissue Sarcoma. J. Cancer Res. Clin. Oncol..

[81] Saerens M., Brusselaers N., Rottey S., Decruyenaere A., Creytens D., Lapeire L. (2021). Immune Checkpoint Inhibitors in Treatment of Soft-Tissue Sarcoma: A Systematic Review and Meta-Analysis. Eur. J. Cancer.

[82] Fridman W.H., Zitvogel L., Sautès-Fridman C., Kroemer G. (2017). The Immune Contexture in Cancer Prognosis and Treatment. Nat. Rev. Clin. Oncol..

[83] Chibon F., Aurias A., Coindre J.-M., Pfeffer U. (2013). Sarcomas Genetics: From Point Mutation to Complex Karyotype, from Diagnosis to Therapies. Cancer Genomics: Molecular Classification, Prognosis and Response Prediction.

[84] Taylor B.S., Barretina J., Maki R.G., Antonescu C.R., Singer S., Ladanyi M. (2011). Advances in Sarcoma Genomics and New Therapeutic Targets. Nat. Rev. Cancer.

[85] Guillou L., Aurias A. (2010). Soft Tissue Sarcomas with Complex Genomic Profiles. Virchows Arch..

[86] Dancsok A.R., Setsu N., Gao D., Blay J.Y., Thomas D., Maki R.G., Nielsen T.O., Demicco E.G. (2019). Expression of Lymphocyte Immunoregulatory Biomarkers in Bone and Soft-Tissue Sarcomas. Mod. Pathol..

[87] Pollack S.M., He Q., Yearley J.H., Emerson R., Vignali M., Zhang Y., Redman M.W., Baker K.K., Cooper S., Donahue B. (2017). T-Cell Infiltration and Clonality Correlate with Programmed Cell Death Protein 1 and Programmed Death-Ligand 1 Expression in Patients with Soft Tissue Sarcomas. Cancer.

[88] Yan L., Wang Z., Cui C., Guan X., Dong B., Zhao M., Wu J., Tian X., Hao C. (2019). Comprehensive Immune Characterization and T-Cell Receptor Repertoire Heterogeneity of Retroperitoneal Liposarcoma. Cancer Sci..

[89] Oike N., Kawashima H., Ogose A., Hatano H., Ariizumi T., Yamagishi T., Murayama Y., Umezu H., Imai C., Hayashi M. (2021). Human Leukocyte Antigen I Is Significantly Downregulated in Patients with Myxoid Liposarcomas. Cancer Immunol. Immunother..

[90] Abeshouse A., Adebamowo C., Adebamowo S.N., Akbani R., Akeredolu T., Ally A., Anderson M.L., Anur P., Appelbaum E.L., Armenia J. (2017). Comprehensive and Integrated Genomic Characterization of Adult Soft Tissue Sarcomas. Cell.

[91] Simon M., Mughal S.S., Horak P., Uhrig S., Buchloh J., Aybey B., Stenzinger A., Glimm H., Fröhling S., Brors B. (2021). Deconvolution of Sarcoma Methylomes Reveals Varying Degrees of Immune Cell Infiltrates with Association to Genomic Aberrations. J. Transl. Med..

[92] Orth M.F., Buecklein V.L., Kampmann E., Subklewe M., Noessner E., Cidre-Aranaz F., Romero-Pérez L., Wehweck F.S., Lindner L., Issels R. (2020). A Comparative View on the Expression Patterns of PD-L1 and PD-1 in Soft Tissue Sarcomas. Cancer Immunol. Immunother..

[93] Melake M., Smith H., Mansfield D., Davies E., Dillon M., Wilkins A., Patin E., Pedersen M., Buus R., Melcher A. (2022). OX40 and 4-1BB Delineate Distinct Immune Profiles in Sarcoma. Oncoimmunology.

[94] Klaver Y., Rijnders M., Oostvogels A., Wijers R., Smid M., Grünhagen D., Verhoef K., Sleijfer S., Lamers C., Debets R. (2020). Differential Quantities of Immune Checkpoint-Expressing CD8 T Cells in Soft Tissue Sarcoma Subtypes. J. Immunother. Cancer.

[95] Smolle M.A., Herbsthofer L., Granegger B., Goda M., Brcic I., Bergovec M., Scheipl S., Prietl B., Pichler M., Gerger A. (2021). T-Regulatory Cells Predict Clinical Outcome in Soft Tissue Sarcoma Patients: A Clinico-Pathological Study. Br. J. Cancer.

[96] Smolle M.A., Herbsthofer L., Goda M., Granegger B., Brcic I., Bergovec M., Scheipl S., Prietl B., El-Heliebi A., Pichler M. (2021). Influence of Tumor-Infiltrating Immune Cells on Local Control Rate, Distant Metastasis, and Survival in Patients with Soft Tissue Sarcoma. Oncoimmunology.

[97] Wunder J.S., Lee M.J., Nam J., Lau B.Y., Dickson B.C., Pinnaduwage D., Bull S.B., Ferguson P.C., Seto A., Gokgoz N. (2020). Osteosarcoma and Soft-Tissue Sarcomas with an Immune Infiltrate Express PD-L1: Relation to Clinical Outcome and Th1 Pathway Activation. Oncoimmunology.

[98] Schroeder B.A., Lafranzo N.A., Lafleur B.J., Gittelman R.M., Vignali M., Zhang S., Flanagan K.C., Rytlewski J., Riolobos L., Schulte B.C. (2021). CD4+ T Cell and M2 Macrophage Infiltration Predict Dedifferentiated Liposarcoma Patient Outcomes. J. Immunother. Cancer.

[99] Minopoli M., Sarno S., Cannella L., Tafuto S., Scognamiglio G., Gallo M., Fazioli F., Azzaro R., Apice G., de Angelis B. (2021). Crosstalk between Macrophages and Myxoid Liposarcoma Cells Increases Spreading and Invasiveness of Tumor Cells. Cancers.

[100] Sorbye S.W., Kilvaer T., Valkov A., Donnem T., Smeland E., Al-Shibli K., Bremnes R.M., Busund L.T. (2012). High Expression of CD20+ Lymphocytes in Soft Tissue Sarcomas Is a Positive Prognostic Indicator. Oncoimmunology.

[101] Judge S.J., Darrow M.A., Thorpe S.W., Gingrich A.A., O’Donnell E.F., Bellini A.R., Sturgill I.R., Vick L.V., Dunai C., Stoffel K.M. (2020). Analysis of Tumor-Infiltrating NK and T Cells Highlights IL-15 Stimulation and TIGIT Blockade as a Combination Immunotherapy Strategy for Soft Tissue Sarcomas. J. Immunother. Cancer.

[102] D’Angelo S.P., Shoushtari A.N., Agaram N.P., Kuk D., Qin L.X., Carvajal R.D., Dickson M.A., Gounder M., Keohan M.L., Schwartz G.K. (2015). Prevalence of Tumor-Infiltrating Lymphocytes and PD-L1 Expression in the Soft Tissue Sarcoma Microenvironment. Hum. Pathol..

[103] Issels R.D., Noessner E., Lindner L.H., Schmidt M., Albertsmeier M., Blay J.Y., Stutz E., Xu Y., Buecklein V., Altendorf-Hofmann A. (2021). Immune Infiltrates in Patients with Localised High-Risk Soft Tissue Sarcoma Treated with Neoadjuvant Chemotherapy without or with Regional Hyperthermia: A Translational Research Program of the EORTC 62961-ESHO 95 Randomised Clinical Trial. Eur. J. Cancer.

[104] Zhu N., Hou J. (2020). Assessing Immune Infiltration and the Tumor Microenvironment for the Diagnosis and Prognosis of Sarcoma. Cancer Cell Int..

[105] Zhang L., Lin W., Zhou Y., Shao F., Gao Y., He J. (2022). A Complement-Related Gene Signature for Predicting Overall Survival and Immunotherapy Efficacy in Sarcoma Patients. Front. Cell Dev. Biol..

[106] Li N., Yuan J., Tian W., Meng L., Liu Y. (2020). T-Cell Receptor Repertoire Analysis for the Diagnosis and Treatment of Solid Tumor: A Methodology and Clinical Applications. Cancer Commun..

[107] Paijens S.T., Vledder A., de Bruyn M., Nijman H.W. (2021). Tumor-Infiltrating Lymphocytes in the Immunotherapy Era. Cell Mol. Immunol..

[108] Tawbi H.A., Burgess M., Bolejack V., van Tine B.A., Schuetze S.M., Hu J., D’Angelo S., Attia S., Riedel R.F., Priebat D.A. (2017). Pembrolizumab in Advanced Soft-Tissue Sarcoma and Bone Sarcoma (SARC028): A Multicentre, Two-Cohort, Single-Arm, Open-Label, Phase 2 Trial. Lancet Oncol..

[109] Chakravarthy A., Furness A., Joshi K., Ghorani E., Ford K., Ward M.J., King E.V., Lechner M., Marafioti T., Quezada S.A. (2018). Pan-Cancer Deconvolution of Tumour Composition Using DNA Methylation. Nat. Commun..

[110] Galon J., Bruni D. (2019). Approaches to Treat Immune Hot, Altered and Cold Tumours with Combination Immunotherapies. Nat. Rev. Drug Discov..

[111] Sharma A., Bode B., Studer G., Moch H., Okoniewski M., Knuth A., von Boehmer L., van den Broek M. (2013). Radiotherapy of Human Sarcoma Promotes an Intratumoral Immune Effector Signature. Clin. Cancer Res..

[112] Snow H., Mitchell C., Hendry S., McKinley M., Byrne D., Ngan S., Chander S., Chu J., Desai J., Bae S. (2021). Characterising the Immune Microenvironment in Liposarcoma, Its Impact on Prognosis and the Impact of Radiotherapy. J. Surg. Oncol..

[113] Zhang S., Kohli K., Graeme Black R., Yao L., Spadinger S.M., He Q., Pillarisetty V.G., Cranmer L.D., van Tine B.A., Yee C. (2019). Systemic Interferon-g Increases MHC Class I Expression and T-Cell Infiltration in Cold Tumors: Results of a Phase 0 Clinical Trial. Cancer Immunol. Res..

[114] Italiano A., Bessede A., Pulido M., Bompas E., Piperno-Neumann S., Chevreau C., Penel N., Bertucci F., Toulmonde M., Bellera C. (2022). Pembrolizumab in Soft-Tissue Sarcomas with Tertiary Lymphoid Structures: A Phase 2 PEMBROSARC Trial Cohort. Nat. Med..

[115] Sharonov G.V., Serebrovskaya E.O., Yuzhakova D.V., Britanova O.V., Chudakov D.M. (2020). B Cells, Plasma Cells and Antibody Repertoires in the Tumour Microenvironment. Nat. Rev. Immunol..

[116] Sautès-Fridman C., Petitprez F., Calderaro J., Fridman W.H. (2019). Tertiary Lymphoid Structures in the Era of Cancer Immunotherapy. Nat. Rev. Cancer.

[117] Tsagozis P., Augsten M., Zhang Y., Li T., Hesla A., Bergh J., Haglund F., Tobin N.P., Ehnman M. (2019). An Immunosuppressive Macrophage Profile Attenuates the Prognostic Impact of CD20-Positive B Cells in Human Soft Tissue Sarcoma. Cancer Immunol. Immunother..

[118] Tseng W.W., Malu S., Zhang M., Chen J., Sim G.C., Wei W., Ingram D., Somaiah N., Lev D.C., Pollock R.E. (2015). Analysis of the Intratumoral Adaptive Immune Response in Well Differentiated and Dedifferentiated Retroperitoneal Liposarcoma. Sarcoma.

[119] Cózar B., Greppi M., Carpentier S., Narni-Mancinelli E., Chiossone L., Vivier E. (2021). Tumor-Infiltrating Natural Killer Cells. Cancer Discov..

[120] Pan Y., Yu Y., Wang X., Zhang T. (2020). Tumor-Associated Macrophages in Tumor Immunity. Front. Immunol..

[121] Dancsok A.R., Gao D., Lee A.F., Steigen S.E., Blay J.Y., Thomas D.M., Maki R.G., Nielsen T.O., Demicco E.G. (2020). Tumor-Associated Macrophages and Macrophage-Related Immune Checkpoint Expression in Sarcomas. Oncoimmunology.

[122] Nabeshima A., Matsumoto Y., Fukushi J., Iura K., Matsunobu T., Endo M., Fujiwara T., Iida K., Fujiwara Y., Hatano M. (2015). Tumour-Associated Macrophages Correlate with Poor Prognosis in Myxoid Liposarcoma and Promote Cell Motility and Invasion via the HB-EGF-EGFR-PI3K/Akt Pathways. Br. J. Cancer.

[123] Sorbye S.W., Kilvaer T.K., Valkov A., Donnem T., Smeland E., Al-Shibli K., Bremnes R.M., Busund L.T. (2012). Prognostic Impact of CD57, CD68, M-CSF, CSF-1R, Ki67 and TGF-Beta in Soft Tissue Sarcomas. BMC Clin. Pathol..

[124] Keung E.Z., Tsai J.W., Ali A.M., Cormier J.N., Bishop A.J., Guadagnolo B.A., Torres K.E., Somaiah N., Hunt K.K., Wargo J.A. (2018). Analysis of the Immune Infiltrate in Undifferentiated Pleomorphic Sarcoma of the Extremity and Trunk in Response to Radiotherapy: Rationale for Combination Neoadjuvant Immune Checkpoint Inhibition and Radiotherapy. Oncoimmunology.

[125] Rutkowski P., Kaminska J., Kowalska M., Ruka W., Steffen J. (2002). Cytokine Serum Levels in Soft Tissue Sarcoma Patients: Correlations with Clinico-Pathological Features and Prognosis. Int. J. Cancer.

[126] Hagi T., Nakamura T., Iino T., Matsubara T., Asanuma K., Matsumine A., Sudo A. (2017). The Diagnostic and Prognostic Value of Interleukin-6 in Patients with Soft Tissue Sarcomas. Sci. Rep..

[127] Nakamura K., Nakamura T., Iino T., Hagi T., Kita K., Asanuma K., Sudo A. (2020). Expression of Interleukin-6 and the Interleukin-6 Receptor Predicts the Clinical Outcomes of Patients with Soft Tissue Sarcomas. Cancers.

[128] Casadei L., Calore F., Creighton C.J., Guescini M., Batte K., Iwenofu O.H., Zewdu A., Braggio D.A., Bill K.L., Fadda P. (2017). Exosome-Derived MiR-25-3p and MiR-92a-3p Stimulate Liposarcoma Progression. Cancer Res..

[129] Kampan N.C., Xiang S.D., McNally O.M., Stephens A.N., Quinn M.A., Plebanski M. (2018). Immunotherapeutic Interleukin-6 or Interleukin-6 Receptor Blockade in Cancer: Challenges and Opportunities. Curr. Med. Chem..

[130] Mazzu Y.Z., Hu Y., Shen Y., Tuschl T., Singer S. (2019). MiR-193b Regulates Tumorigenesis in Liposarcoma Cells via PDGFR, TGFβ, and Wnt Signaling. Sci. Rep..

[131] Jiang Y., Chen M., Nie H., Yuan Y. (2019). PD-1 and PD-L1 in Cancer Immunotherapy: Clinical Implications and Future Considerations. Hum. Vaccin. Immunother..

[132] Puhr H.C., Ilhan-Mutlu A. (2019). New Emerging Targets in Cancer Immunotherapy: The Role of LAG3. ESMO Open.

[133] He Y., Cao J., Zhao C., Li X., Zhou C., Hirsch F.R. (2018). TIM-3, a Promising Target for Cancer Immunotherapy. Onco Targets Ther..

[134] Shiravand Y., Khodadadi F., Kashani S.M.A., Hosseini-Fard S.R., Hosseini S., Sadeghirad H., Ladwa R., O’Byrne K., Kulasinghe A. (2022). Immune Checkpoint Inhibitors in Cancer Therapy. Curr. Oncol..

[135] Movva S., Wen W., Chen W., Millis S.Z., Gatalica Z., Reddy S., von Mehren M., van Tine B.A. (2015). Multi-Platform Profiling of over 2000 Sarcomas: Identification of Biomarkers and Novel Therapeutic Targets. Oncotarget.

[136] Torabi A., Amaya C.N., Wians F.H., Bryan B.A. (2017). PD-1 and PD-L1 Expression in Bone and Soft Tissue Sarcomas. Pathology.

[137] Kim J.R., Moon Y.J., Kwon K.S., Bae J.S., Wagle S., Kim K.M., Park H.S., Lee H., Moon W.S., Chung M.J. (2013). Tumor Infiltrating PD1-Positive Lymphocytes and the Expression of PD-L1 Predict Poor Prognosis of Soft Tissue Sarcomas. PLoS ONE.

[138] Miyake M., Oda Y., Nishimura N., Morizawa Y., Ohnishi S., Hatakeyama K., Fujii T., Hori S., Gotoh D., Nakai Y. (2020). Integrative Assessment of Clinicopathological Parameters and the Expression of PD-L1, PD-L2 and PD-1 in Tumor Cells of Retroperitoneal Sarcoma. Oncol. Lett..

[139] (2022). FDA Approves Anti-LAG3 Checkpoint. Nat. Biotechnol..

[140] Que Y., Fang Z., Guan Y., Xiao W., Xu B., Zhao J., Chen H., Zhang X., Zeng M., Liang Y. (2019). LAG-3 Expression on Tumor-Infiltrating T Cells in Soft Tissue Sarcoma Correlates with Poor Survival. Cancer Biol. Med..

[141] Dufresne A., Lesluyes T., Ménétrier-Caux C., Brahmi M., Darbo E., Toulmonde M., Italiano A., Mir O., le Cesne A., le Guellec S. (2020). Specific Immune Landscapes and Immune Checkpoint Expressions in Histotypes and Molecular Subtypes of Sarcoma. Oncoimmunology.

[142] Que Y., Xiao W., Guan Y.X., Liang Y., Yan S.M., Chen H.Y., Li Q.Q., Xu B.S., Zhou Z.W., Zhang X. (2017). PD-L1 Expression Is Associated with FOXP3+ Regulatory T-Cell Infiltration of Soft Tissue Sarcoma and Poor Patient Prognosis. J. Cancer.

[143] Zheng B., Wang J., Cai W., Lao I., Shi Y., Luo X., Yan W. (2019). Changes in the Tumor Immune Microenvironment in Resected Recurrent Soft Tissue Sarcomas. Ann. Transl. Med..

[144] Budczies J., Mechtersheimer G., Denkert C., Klauschen F., Mughal S.S., Chudasama P., Bockmayr M., Jöhrens K., Endris V., Lier A. (2017). PD-L1 (CD274) Copy Number Gain, Expression, and Immune Cell Infiltration as Candidate Predictors for Response to Immune Checkpoint Inhibitors in Soft-Tissue Sarcoma. Oncoimmunology.

[145] Roulleaux Dugage M., Nassif E.F., Italiano A., Bahleda R. (2021). Improving Immunotherapy Efficacy in Soft-Tissue Sarcomas: A Biomarker Driven and Histotype Tailored Review. Front. Immunol..

[146] Tan A.C., Bagley S.J., Wen P.Y., Lim M., Platten M., Colman H., Ashley D.M., Wick W., Chang S.M., Galanis E. (2021). Systematic Review of Combinations of Targeted or Immunotherapy in Advanced Solid Tumors. J. Immunother. Cancer.

[147] Sadeghi Rad H., Monkman J., Warkiani M.E., Ladwa R., O’Byrne K., Rezaei N., Kulasinghe A. (2021). Understanding the Tumor Microenvironment for Effective Immunotherapy. Med. Res. Rev..

[148] Ozaniak A., Vachtenheim J., Lischke R., Bartunkova J., Strizova Z. (2021). Novel Insights into the Immunotherapy of Soft Tissue Sarcomas: Do We Need a Change of Perspective?. Biomedicines.

[149] Mizukoshi E., Kaneko S. (2019). Telomerase-Targeted Cancer Immunotherapy. Int. J. Mol. Sci..

[150] Parkhurst M.R., Riley J.P., Igarashi T., Li Y., Robbins P.F., Rosenberg S.A. (2004). Immunization of Patients with the HTERT:540-548 Peptide Induces Peptide-Reactive T Lymphocytes That Do Not Recognize Tumors Endogenously Expressing Telomerase. Clin. Cancer Res..

[151] Vafaei S., Zekiy A.O., Khanamir R.A., Zaman B.A., Ghayourvahdat A., Azimizonuzi H., Zamani M. (2022). Combination Therapy with Immune Checkpoint Inhibitors (ICIs); a New Frontier. Cancer Cell Int..

[152] D’Angelo S.P., Mahoney M.R., van Tine B.A., Atkins J., Milhem M.M., Jahagirdar B.N., Antonescu C.R., Horvath E., Tap W.D., Schwartz G.K. (2018). Nivolumab with or without Ipilimumab Treatment for Metastatic Sarcoma (Alliance A091401): Two Open-Label, Non-Comparative, Randomised, Phase 2 Trials. Lancet Oncol..

[153] Márquez-Rodas I., Longo F., Rodriguez-Ruiz M.E., Calles A., Ponce S., Jove M., Rubio-Viqueira B., Perez-Gracia J.L., Gómez-Rueda A., López-Tarruella S. (2020). Intratumoral Nanoplexed Poly I:C BO-112 in Combination with Systemic Anti-PD-1 for Patients with Anti-PD-1-Refractory Tumors. Sci. Transl. Med..

[154] Wagner M.J., Zhang Y., Cranmer L.D., Loggers E.T., Black G., McDonnell S., Maxwell S., Johnson R., Moore R., de Viveiros P.H. (2022). A Phase 1/2 Trial Combining Avelumab and Trabectedin for Advanced Liposarcoma and Leiomyosarcoma. Clin. Cancer Res..

[155] Smith B.D., Kaufman M.D., Wise S.C., Ahn Y.M., Caldwell T.M., Leary C.B., Lu W.P., Tan G., Vogeti L., Vogeti S. (2021). Vimseltinib: A Precision CSF1R Therapy for Tenosynovial Giant Cell Tumors and Diseases Promoted by Macrophages. Mol. Cancer Ther..

[156] Chawla S.P., van Tine B.A., Pollack S.M., Ganjoo K.N., Elias A.D., Riedel R.F., Attia S., Choy E., Okuno S.H., Agulnik M. (2022). Phase II Randomized Study of CMB305 and Atezolizumab Compared with Atezolizumab Alone in Soft-Tissue Sarcomas Expressing NY-ESO-1. J. Clin. Oncol..

[157] Osgood C.L., Chuk M.K., Theoret M.R., Huang L., He K., Her L., Keegan P., Pazdur R. (2017). FDA Approval Summary: Eribulin for Patients with Unresectable or Metastatic Liposarcoma Who Have Received a Prior Anthracycline-Containing Regimen. Clin. Cancer Res..

[158] Pollack S.M., Jungbluth A.A., Hoch B.L., Farrar E.A., Bleakley M., Schneider D.J., Loggers E.T., Rodler E., Eary J.F., Conrad E.U. (2012). NY-ESO-1 Is a Ubiquitous Immunotherapeutic Target Antigen for Patients with Myxoid/Round Cell Liposarcoma. Cancer.

[159] Iura K., Kohashi K., Ishii T., Maekawa A., Bekki H., Otsuka H., Yamada Y., Yamamoto H., Matsumoto Y., Iwamoto Y. (2017). MAGEA4 Expression in Bone and Soft Tissue Tumors: Its Utility as a Target for Immunotherapy and Diagnostic Marker Combined with NY-ESO-1. Virchows Arch..

[160] Jo U., Roh J., Song M.J., Cho K.-J., Kim W., Song J.S. (2022). NY-ESO-1 as a Diagnostic and Prognostic Marker for Myxoid Liposarcoma. Am. J. Transl. Res..

[161] Jiang Z., Jiang X., Chen S., Lai Y., Wei X., Li B., Lin S., Wang S., Wu Q., Liang Q. (2017). Anti-GPC3-CAR T Cells Suppress the Growth of Tumor Cells in Patient-Derived Xenografts of Hepatocellular Carcinoma. Front. Immunol..

